# Genome‐Wide CRISPR Screen Identifies a microRNA Orchestrating Pleiotropic Resistance to Targeted Therapy and T Cell Immunity in Melanoma

**DOI:** 10.1002/advs.202515158

**Published:** 2026-05-26

**Authors:** Zhao Wang, Haotian Liu, Xueqi Wang, Jianjin Teng, Zihan Zheng, Jingya Zhang, Wanyi Xiao, Qian Liang, Jiaxin Li, Xiaomeng Jia, Xin Feng, Hongzhang Cui, Menghan Luo, Tielong Yang, Liuxing Wu, Ke Zhao, Weilong Yang, Mulin Jun Li, Dandan Huang, Jilong Yang

**Affiliations:** ^1^ Department of Pharmacology State Key Laboratory of Experimental Hematology The Province and Ministry Co‐sponsored Collaborative Innovation Center for Medical Epigenetics Tianjin Key Laboratory of Inflammation Biology School of Basic Medical Sciences Tianjin Medical University Cancer Institute and Hospital Tianjin Medical University Tianjin China; ^2^ Oujiang Laboratory Wenzhou Medical University Wenzhou China; ^3^ Department of Bone and Soft Tissue Tumor Tianjin Medical University Cancer Institute and Hospital Key Laboratory of Cancer Prevention and Therapy State Key Laboratory of Druggability Evaluation and Systematic Translational Medicine National Clinical Research Center for Cancer Tianjin China; ^4^ Department of Bioinformatics School of Basic Medical Sciences Tianjin Medical University Tianjin China; ^5^ Guangzhou Women and Children's Medical Center Guangzhou Medical University Guangzhou China; ^6^ Scientific Research Center Wenzhou Medical University Wenzhou China

**Keywords:** CRISPR screen, hippo signaling, miR‐18a, melanoma, targeted therapy resistance, T cell immunity, THBS1–CD47 axis

## Abstract

Acquired resistance to both targeted therapies and immunotherapies in cancer presents major clinical challenges, yet the molecular mechanisms underlying cross‐resistance remain poorly understood. We hypothesized that loss of specific microRNAs (miRNAs) could potentiate melanoma resistance to both targeted drugs and CD8^+^ T cell‐mediated cytotoxicity. Through genome‐wide miRNA CRISPR knockout screening integrated with cellular models, longitudinal clinical samples, and in vivo experiments, we identified miR‐18a as a pivotal upstream regulator of pleiotropic resistance in melanoma. We show that miR‐18a deficiency drives resistance through two distinct mechanisms: derepressing AJUBA‐regulated Hippo signaling during MAPK inhibition, and enhancing THBS1–CD47 interactions that impair the immunological synapse between tumor cells and CD8^+^ T cells. Furthermore, hnRNP A1 plays an essential role in modulating miR‐18a expression, thereby mediating cross‐resistance. These findings suggest that targeting non‐coding RNA vulnerabilities may represent a promising therapeutic strategy to overcome complex resistance mechanisms and improve clinical outcomes in melanoma.

## Graphical abstract

A genome‐wide microRNA CRISPR screen identifies miR‐18a as a master regulator of cross‐resistance in melanoma. Loss of miR‐18a activates the AJUBA–YAP/Hippo axis to confer BRAFi resistance and enhances THBS1–CD47 interaction to impair CD8^+^ T cell immunity. hnRNP A1 is identified as an upstream regulator of miR‐18a processing. Multi‐omic, single‐cell, in vivo, and clinical data collectively support these findings.

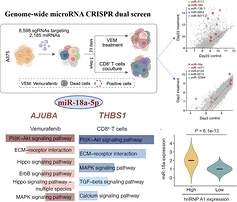

## 1 Introduction

Melanoma treatment currently faces formidable clinical challenges, driven largely by resistance to BRAF/MEK‐targeted therapies and CTLA‐4/PD‐1/PD‐L1 immunotherapies [1, 2]. Although sequential and combinatorial regimens may delay or overcome resistance, patients who relapse following initial therapy often exhibit diminished responses to subsequent treatments, frequently leading to irreversible disease progression and death [3, 4, 5]. Furthermore, intratumoral heterogeneity and clonal evolution may confer resistance not only to current therapies but also to future interventions, complicating therapeutic decision‐making [6, 7]. A critical knowledge gap persists regarding the molecular mechanisms underlying this cross‐resistance, particularly those operating upstream of well‐characterized pathways.

Molecular mechanisms underlying pleiotropic resistance in melanoma treatment are being progressively elucidated. Profound tumor cell plasticity and intratumoral heterogeneity serve as major drivers of this resistance, frequently amplified by adaptive changes induced by selection pressures from prior therapies [2, 8]. Key mediators of cross‐resistance have been identified, including reactivation of the MAPK pathway and dysregulation of Hippo signaling [9, 10]. Moreover, alterations in the tumor microenvironment—including CD8^+^ T cell exhaustion [11], accumulation of myeloid‐derived suppressor cells [12], and deficiencies in specific dendritic cell subsets [13]—establish immunosuppressive niches that compromise the efficacy of subsequent immune and combination therapies. However, most research has centered on functional and phenotypic characterization to identify critical signaling axes and master regulators, with limited investigation into the causal molecular entities acting upstream.

MicroRNAs (miRNAs), exemplified by the well‐characterized miR‐17‐92 cluster and miR‐21 [14, 15, 16], are emerging as pivotal regulators of tumor development and drug resistance. Given their ability to modulate multiple signaling pathways and biological processes, specific miRNAs may undergo selection during tumor therapy, thereby promoting resistance to subsequent treatments and contributing to pleiotropic resistance. We hypothesized that loss of a specific miRNA could confer melanoma cell resistance to both the FDA‐approved BRAF inhibitor vemurafenib (BRAFi, VEM) and CD8^+^ T cell‐mediated cytotoxicity. To test this, we employed a forward genetics approach using genome‐wide miRNA CRISPR knockout (KO) screening. This systematic exploration yielded the unexpected discovery that miR‐18a plays a critical role in mediating resistance to both targeted treatment and CD8^+^ T cell cytotoxicity. Through integration of resistant cell models, longitudinal clinical samples, and in vivo experiments, we identified miR‐18a as a candidate regulator associated with poor outcomes in both targeted and immune therapies for melanoma. Our findings further illuminate how miR‐18a loss drives dysfunction of the Hippo and TGF‐β pathways, sculpting the complex landscape of pleiotropic resistance in melanoma. These results highlight a potential non‐coding RNA vulnerability that could be therapeutically targeted to overcome pleiotropic drug resistance and improve clinical outcomes in cancer.

## 2 Results

### 2.1 Genome‐Wide CRISPR KO Uncovers miR‐18a as a Key Mediator of Dual Resistance to VEM and CD8^+^ T Cells in Melanoma

**FIGURE 1:**
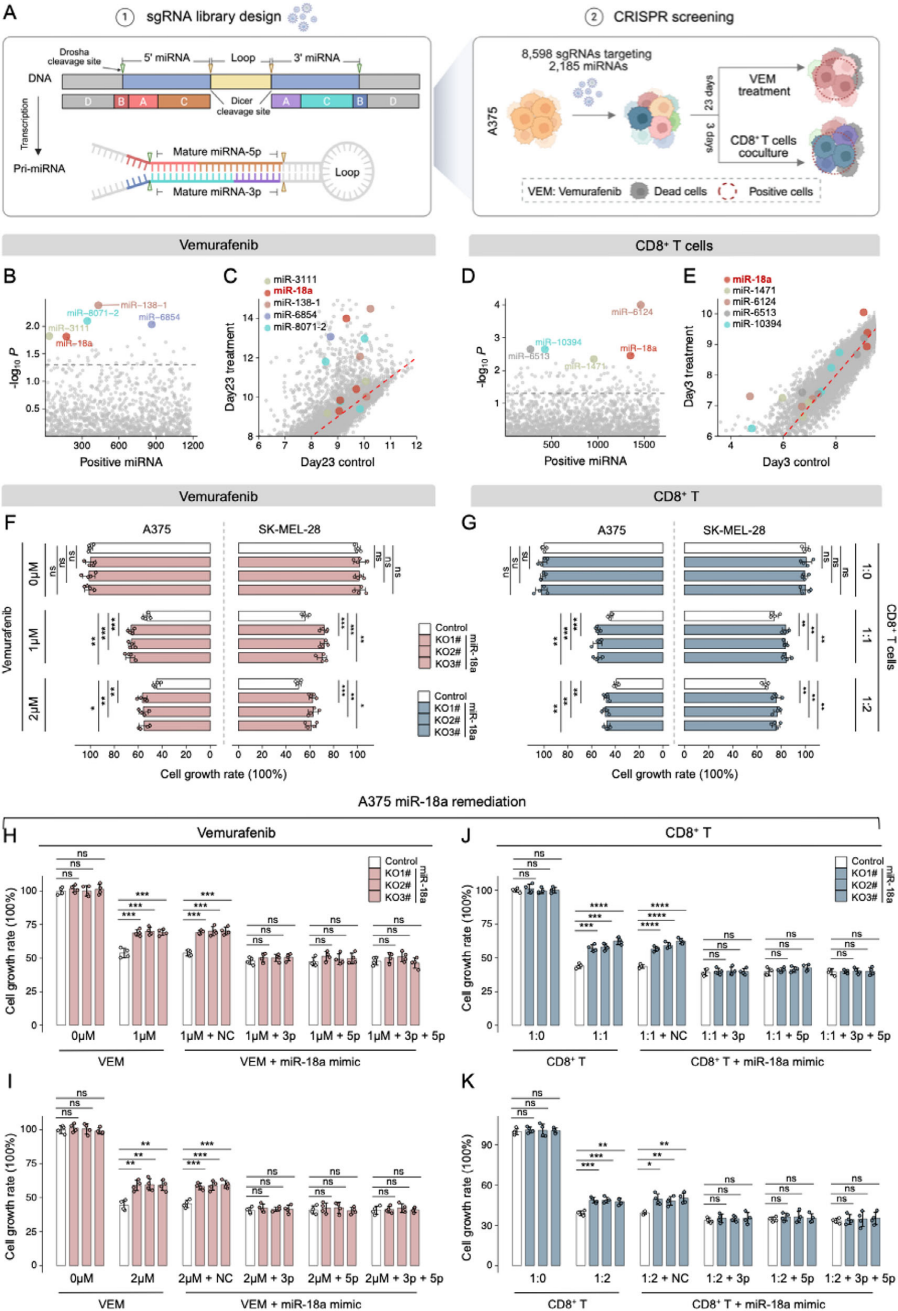
miR‐18a mediates melanoma resistance to vemurafenib and CD8^+^ T cell cytotoxicity. (A) Schematic of CRISPR KO screening strategy to identify miRNAs conferring resistance to vemurafenib (VEM) and CD8^+^ T cell cytotoxicity in melanoma cells. Left: sgRNA library design showing Region A (mature miRNA seed), Region B (Drosha cleavage sites), Region C (mature miRNA non‐seed region and Dicer cleavage site), and Region D (distal pre‐miRNA regions). Right: screening workflow using A375‐Cas9 cells treated with VEM (23 days) and co‐cultured with CD8^+^ T cells (3 days). (B) Top 5 candidate miRNAs conferring VEM resistance under positive selection. (C) Scatterplot showing enrichment (log_2_ count) of sgRNAs targeting miRNAs post‐VEM treatment, highlighting sgRNAs for top 5 candidates. (D) Top 5 candidate miRNAs conferring resistance to CD8^+^ T cell‐mediated cytotoxicity. (E) Scatterplot showing enrichment (log_2_ count) of sgRNAs targeting miRNAs following CD8^+^ T cell co‐culture, highlighting sgRNAs for top 5 candidates. (F) Cell proliferation analysis (24 h) following VEM treatment (0, 1, and 2 µm) in miR‐18a knockout (KO) A375 and SK‐MEL‐28 melanoma cells. (G) Cell proliferation under 24 h CD8^+^ T cell co‐culture (target‐to‐effector [T:E] ratios 1:0, 1:1, and 1:2) in miR‐18a KO A375 and SK‐MEL‐28 cells. (H, I) Restoration of VEM sensitivity by miR‐18a mimics at 1 µm (H) and 2 µm (I). (J, K) Restoration of CD8^+^ T cell cytotoxicity by miR‐18a mimics at T:E ratios 1:1 (J) and 1:2 (K). NC: scrambled control; 3p/5p: miR‐18a‐3p/5p mimics; Control: A375‐Cas9 or SK‐MEL‐28‐Cas9 cells. Data represent means ± SD; *n* = 4 biologically independent samples for panels F–K. Unpaired two‐tailed Student's *t*‐test is used to calculate *P*‐values in panels F–K: *, *P* < 0.05; **, *P* < 0.01; ***, *P* < 0.001; ****, *P* < 0.0001; ns, not significant.

We systematically investigated miRNA functions in melanoma therapeutic responses by conducting two parallel large‐scale CRISPR screening experiments targeting both VEM treatment and CD8^+^ T cell‐mediated killing. Existing genome‐wide miRNA CRISPR KO libraries were either embedded within broader protein‐coding gene libraries or lacked comprehensive coverage of the latest known miRNA repertoire (pre‐miRNAs) [17, 18]. Building upon design principles from an earlier TALEN‐based miRNA KO library [19], we developed a refined CRISPR/Cas9 single guide RNA (sgRNA) library targeting critical regions within the secondary structures of all pre‐miRNAs cataloged in miRBase v21 [20] and FANTOM5 [21]. This library comprises 8598 sgRNAs targeting 2185 miRNAs, with an average of approximately four sgRNAs per miRNA (Table S1), representing a substantial advancement in both miRNA coverage breadth and targeting precision of functionally critical pre‐miRNA regions compared to previous libraries (Figure S1A). Using this comprehensive genome‐wide library and the CRISPR/Cas9 KO screening platform, we designed two independent screens in BRAF V600E‐mutant melanoma A375 cells to systematically identify miRNAs whose loss confers resistance to VEM treatment and CD8^+^ T cell cytotoxicity (Figure 1A).

Briefly, the melanoma A375 cell line, which is sensitive to both BRAF and MEK inhibitors (BRAFi/MEKi) and T cell‐mediated cytotoxicity, was engineered to stably express CRISPR/Cas9 (A375‐Cas9 cells). These cells underwent lentiviral transduction with a pooled sgRNA library at a low multiplicity of infection (MOI = 0.3). Following transduction, cells were subjected to puromycin selection for one week, then exposed to several days of selection either with or without VEM treatment, or co‐cultured with or without stimulated CD8^+^ T cells isolated from healthy donors. CD8^+^ T cells were non‐specifically activated using IL‐2 and anti‐CD3/CD28 antibodies (see Methods for details). This approach enabled identification of miRNAs that modulate resistance to targeted therapies or T cell‐mediated cytotoxicity (Figure 1A). Integrated sgRNA sequences were PCR‐amplified and subjected to next‐generation sequencing to assess enrichment by comparing treated versus untreated conditions, while accounting for potential sgRNA effects on cellular proliferation and viability. The MAGeCK algorithm [22] was employed to identify positively selected sgRNAs (see Methods for details). Replicate screens demonstrated high reproducibility (*r* ≥ 0.9, Spearman's correlation; Figure S1B). Ultimately, 38 and 76 candidate pre‐miRNAs showing nominal statistical significance were prioritized as putative regulators of resistance to VEM treatment or CD8^+^ T cell cytotoxicity, respectively (Tables S2–S5). However, the biological functions of these candidate pre‐miRNAs remain to be systematically validated through rigorous functional experiments.

Positive selection analysis revealed that miR‐18a was concurrently enriched in both the VEM treatment and CD8^+^ T cell killing screens (Figure 1B–E), suggesting that miR‐18a may orchestrate dual resistance to targeted therapy and T cell immunity in melanoma through an unknown mechanism. Although miR‐18a has been extensively implicated in oncogenesis, progression, metastasis, and drug resistance across various cancers, its role as either a tumor promoter or suppressor remains context‐dependent and controversial [23, 24]. To validate the CRISPR screen results and investigate whether miR‐18a loss could induce context‐specific resistance under VEM treatment or CD8^+^ T cell co‐culture conditions, we generated three independent miR‐18a KO monoclonal lines in each of two BRAF V600E‐mutant melanoma cell lines (A375‐Cas9 and SK‐MEL‐28‐Cas9) (Figure S1C–F). Consistent with the CRISPR screen results, miR‐18a depletion conferred resistance to VEM treatment at concentrations of 1 and 2 µm across 24, 48, and 72 h time points (Figure 1F and Figure S1G,H). Furthermore, miR‐18a depletion significantly impaired T cell‐mediated killing of melanoma cells at T:E ratios of 1:1 and 1:2 (Figure 1G). Dose‐response analyses demonstrated that both A375 and SK‐MEL‐28 KO clones exhibited sustained resistance to VEM and T cell cytotoxicity across broader ranges of drug concentrations and T cell ratios compared to unedited control cells (Figure S2A,B). This resistance persisted even when cells were treated with an FDA‐approved MEK inhibitor (MEKi, trametinib) or ERK1/2 kinase inhibitor (ulixertinib), either alone or in combination with VEM (Figure S2C,D). Importantly, reintroduction of mature miR‐18a‐5p, miR‐18a‐3p, or both arms substantially restored sensitivity to VEM and T cell cytotoxicity (Figure 1H–K). Additionally, miR‐18a loss conferred other resistance‐associated phenotypes during treatment; under VEM exposure, both A375 and SK‐MEL‐28 KO clones displayed enhanced clonogenic capacity and reduced apoptosis compared to control cells (Figure S2E–J). We also found that, across CCLE melanoma cell lines, higher miR‐18a‐5p levels were associated with greater sensitivity to dabrafenib, RAF265, and regorafenib (although vemurafenib response data were not available) (Figure S3). Collectively, these findings support a role for miR‐18a as a critical regulator of melanoma cell responses to both VEM treatment and CD8^+^ T cell‐mediated cytotoxicity.

### 2.2 Dysregulated miR‐18a is Associated with Poor Outcomes in Both Targeted and Immune Therapies for Melanoma

**FIGURE 2:**
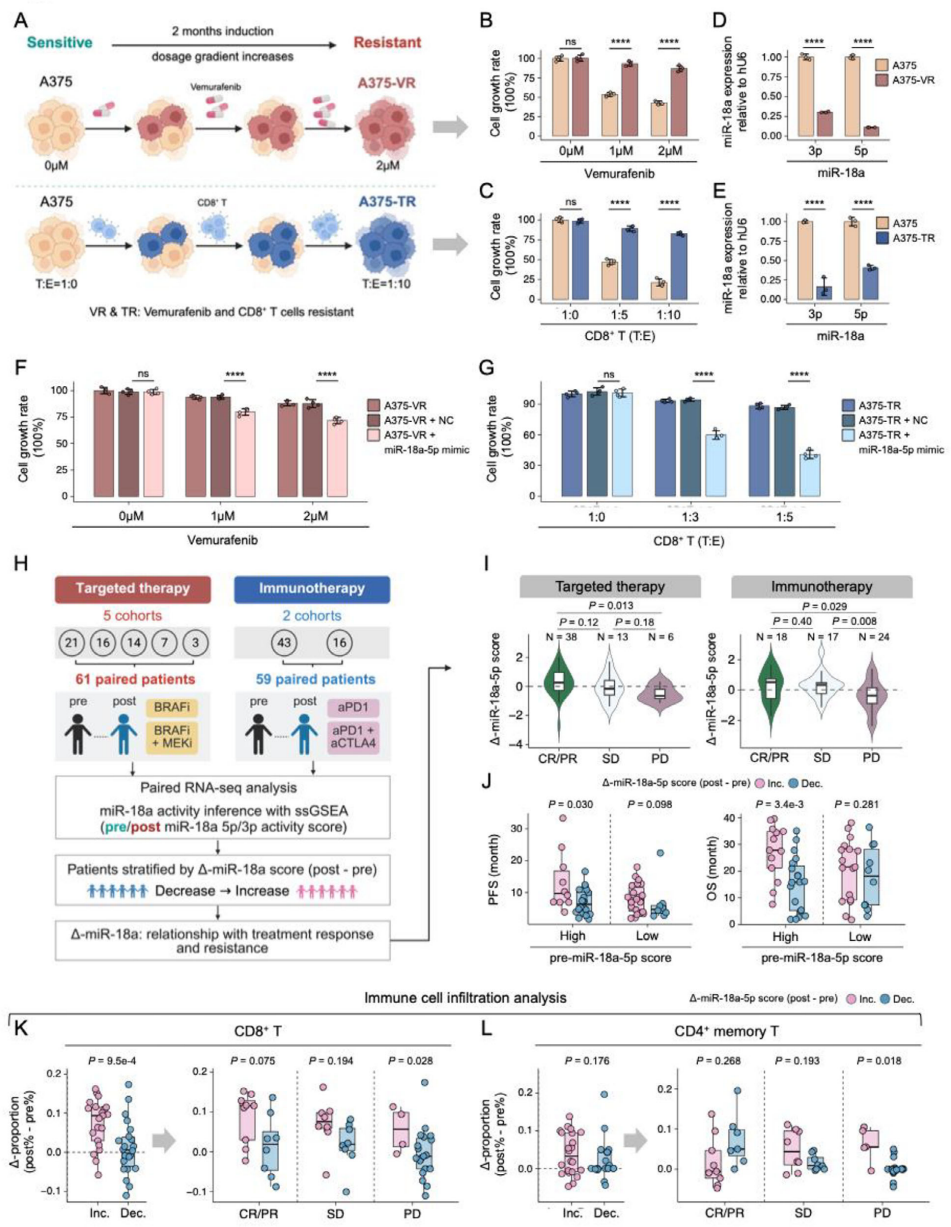
Association of miR‐18a downregulation with resistance to targeted and immune therapies in melanoma. (A) Diagram depicting development of VEM‐resistant (A375‐VR) and CD8^+^ T cell cytotoxicity‐resistant (A375‐TR) melanoma cell lines. (B) Cell proliferation in A375 (control) and A375‐VR cells upon VEM treatment. (C) Cell proliferation of A375 (control) and A375‐TR cells co‐cultured with CD8^+^ T cells at specified T:E ratios. (D, E) miR‐18a‐3p and miR‐18a‐5p expression in A375‐VR (D) and A375‐TR (E) cells relative to parental A375 controls. (F) Cell proliferation of A375‐VR cells transfected with miR‐18a‐5p mimics upon VEM treatment. (G) Cell proliferation of A375‐TR cells transfected with miR‐18a‐5p mimics co‐cultured with CD8^+^ T cells at the indicated T:E ratios. NC: scrambled mimic control. (H) Schematic illustrating Δ‐miR‐18a score calculation and patient stratification (61 targeted therapy, 59 immunotherapy patients). (I) Violin plots showing miR‐18a‐5p score changes in paired samples from patients receiving MAPKi‐targeted therapy or anti‐PD‐1/CTLA‐4 treatment, categorized by RECIST response (CR: complete response, PR: partial response, SD: stable disease, PD: progressive disease). (J) Progression‐free survival (PFS) in targeted therapy cohort and overall survival (OS) in immunotherapy cohort, stratified by pre‐miR‐18a‐5p score and Δ‐miR‐18a‐5p changes (Δ = post‐treatment minus pre‐treatment). (K, L) Changes in CD8^+^ T cells (K) and CD4^+^ memory T cells (L) and correlations with Δ‐miR‐18a‐5p changes. Data represent means ± SD; *n* = 3 biologically independent samples for panels D and E, and *n* = 4 for panels B, C, F, and G. Unpaired two‐tailed Student's *t*‐test is used to calculate *P*‐values in panels B–G: ****, *P* < 0.0001; ns, not significant.

To investigate the role of miR‐18a in drug resistance and immune tolerance, and its potential clinical relevance in melanoma treatment, A375 cells, initially sensitive to VEM and CD8^+^ T cell‐mediated cytotoxicity, were subjected to gradually increasing selective pressures. Specifically, cells were exposed to escalating concentrations of VEM (50 nm–2 µm, with incremental increases every two weeks over two months) or progressively increasing T:E ratios (from 1:1 to 1:10, with alternating cycles of T cell cytotoxic treatment and recovery every two days over the same timeframe) (Figure 2A). This prolonged selection generated VEM‐resistant (A375‐VR) and CD8^+^ T cell cytotoxicity‐resistant (A375‐TR) cell lines, both exhibiting stable resistance phenotypes (Figure 2B,C). Notably, both miR‐18a‐5p and miR‐18a‐3p were significantly downregulated in A375‐VR and A375‐TR cells (Figure 2D,E). Furthermore, reconstitution of miR‐18a restored VEM sensitivity in A375‐VR cells and enhanced susceptibility to CD8^+^ T cell‐mediated cytotoxicity in A375‐TR cells (Figure 2F,G). These findings from induced cellular models suggest that loss of miR‐18a expression may represent a common acquired mechanism of melanoma resistance to both VEM and T cell‐mediated cytotoxicity, warranting further investigation in clinical cohorts.

Integrated omics and clinical data analysis of melanoma samples revealed that the genomic region harboring the miR‐17‐92 cluster, which includes miR‐18a and is encoded by the host gene *MIR17HG*, does not exhibit notable recurrent point mutations (Figure S4A). Further analysis of copy‐number alterations at the *MIR17HG* locus showed that patients with normal copy numbers versus those with copy‐number alterations demonstrated no significant differences in progression‐free survival (PFS) following targeted or immunotherapies (Figure S4B). Beyond genomic studies, extensive clinical research has generated numerous paired pre‐ and post‐treatment tumor samples with outcome data from targeted [6, 25, 26, 27, 28] and immune therapies [29, 30], providing a foundation for exploring the clinical relevance of miR‐18a and its specific targets. Although gene expression profiling is prevalent, these paired samples typically lack miRNA expression data. To elucidate miR‐18a's role in treatment resistance, we performed RNA‐seq on various A375 cell configurations: A375‐Cas9, miR‐18a KO, VEM‐treated, and CD8^+^ T cell‐exposed conditions, with and without miR‐18a KO (Figure S5A and Tables S6 and S7). By integrating differential gene expression analysis with miR‐18a‐5p/3p target prediction (Tables S8 and S9), we identified context‐specific target genes under different treatment exposures (Figure S5B,C and Tables S10–S13). Using RNA‐seq data from clinical samples, we calculated single‐sample Gene Set Enrichment Analysis (ssGSEA) scores, termed miR‐18a scores, reflecting the miR‐18a‐5p or ‐3p signatures in specific treatment contexts (see Methods for details, Figure 2H).

Next, we integrated longitudinal RNA‐seq and clinical data from 120 paired samples obtained from melanoma patients enrolled in seven therapeutic cohorts. These patients received either BRAFi/MEKi (61 paired samples) [6, 25, 26, 27, 28] or immune checkpoint inhibitors (ICIs, 59 paired samples) [29, 30]. This comprehensive analysis was designed to systematically interrogate the clinical relevance of miR‐18a in therapeutic responses and patient prognoses (Figure 2H; Figure S6A–C). We calculated pre‐ and post‐treatment miR‐18a‐5p/3p scores, along with their differential value (∆‐miR‐18a scores, defined as post‐treatment minus pre‐treatment), and systematically explored associations between ∆‐miR‐18a scores and patient responses to BRAFi/MEKi or anti‐PD‐1/CTLA‐4 treatments, as well as survival outcomes. Due to limited sample sizes, some response categories were merged, with ‘Complete Response’ and ‘Partial Response’ combined as CR/PR (Figure 2I,J). Our findings revealed that patients with poorer treatment responses exhibited more pronounced declines in miR‐18a‐5p/3p scores. This suggests that loss of miR‐18a expression may be associated with poor clinical outcomes, consistent with observations in our melanoma cell line models (Figure 2I and Figure S6D–G). Furthermore, when patients were stratified according to changes in miR‐18a‐5p scores (Δ‐miR‐18a‐5p), the association with clinical outcome was more evident in the subgroup with high pre‐treatment miR‐18a‐5p levels. In this subgroup, patients with increased Δ‐miR‐18a‐5p showed relatively better survival than those with decreased Δ‐miR‐18a‐5p in both the targeted therapy and immunotherapy cohorts (Figure 2J). Similar trends were also observed for Δ‐miR‐18a‐3p (Figure S7A,B). Additional sensitivity analyses incorporating available covariates, including treatment, sex, and metastatic stage, showed generally consistent directional effects, although not all models remained statistically significant (Figure S7C–F). Collectively, these findings point to a possible association between therapy‐associated changes in miR‐18a expression and clinical outcome, which warrants further validation in larger, well‐annotated cohorts.

Transcriptomic profiling of longitudinal tumor samples during therapeutic intervention enabled the inference of dynamic alterations in tumor‐infiltrating immune cell composition. In the immunotherapy cohort, we observed significant correlations between ∆‐miR‐18a scores and changes in the proportions of key immune cell types (∆‐proportion, defined as post‐treatment minus pre‐treatment). Notably, ∆‐proportion of CD8^+^ T cells showed the strongest positive correlation with ∆‐miR‐18a‐5p/3p scores (*r *= 0.32, *P *= 0.014 for 5p; *r *= 0.40, *P *= 0.0023 for 3p), while ∆‐proportion of M2 macrophages exhibited a significant negative correlation with ∆‐miR‐18a‐5p scores (*r* = −0.26, *P* = 0.046) (Figure S8A and Tables S18 and S19). Importantly, patients exhibiting declines in miR‐18a‐5p scores during immunotherapy also displayed reduced or stabilized CD8^+^ T cell proportions (Figure 2K). Although no significant overall differences were observed for CD4^+^ memory T cells and M1 macrophages, significant differences emerged within the PD subgroup (Figure 2L and Figure S8B). No significant associations were found between M2 macrophages and response categories (Figure S8C), likely attributable to limited subgroup sample sizes. Similar trends were observed for Δ‐miR‐18a‐3p and immune cell proportions (Figure S8D–G). These findings suggest that miR‐18a loss during immunotherapy may contribute to CD8^+^ T cell exhaustion and establishment of an immunosuppressive tumor microenvironment, underscoring its role in reshaping the immune landscape in melanoma.

### 2.3 Loss of miR‐18a is Associated with Altered Tumor‐Intrinsic Resistance Pathways in Both Cellular Models and Clinical Samples

**FIGURE 3:**
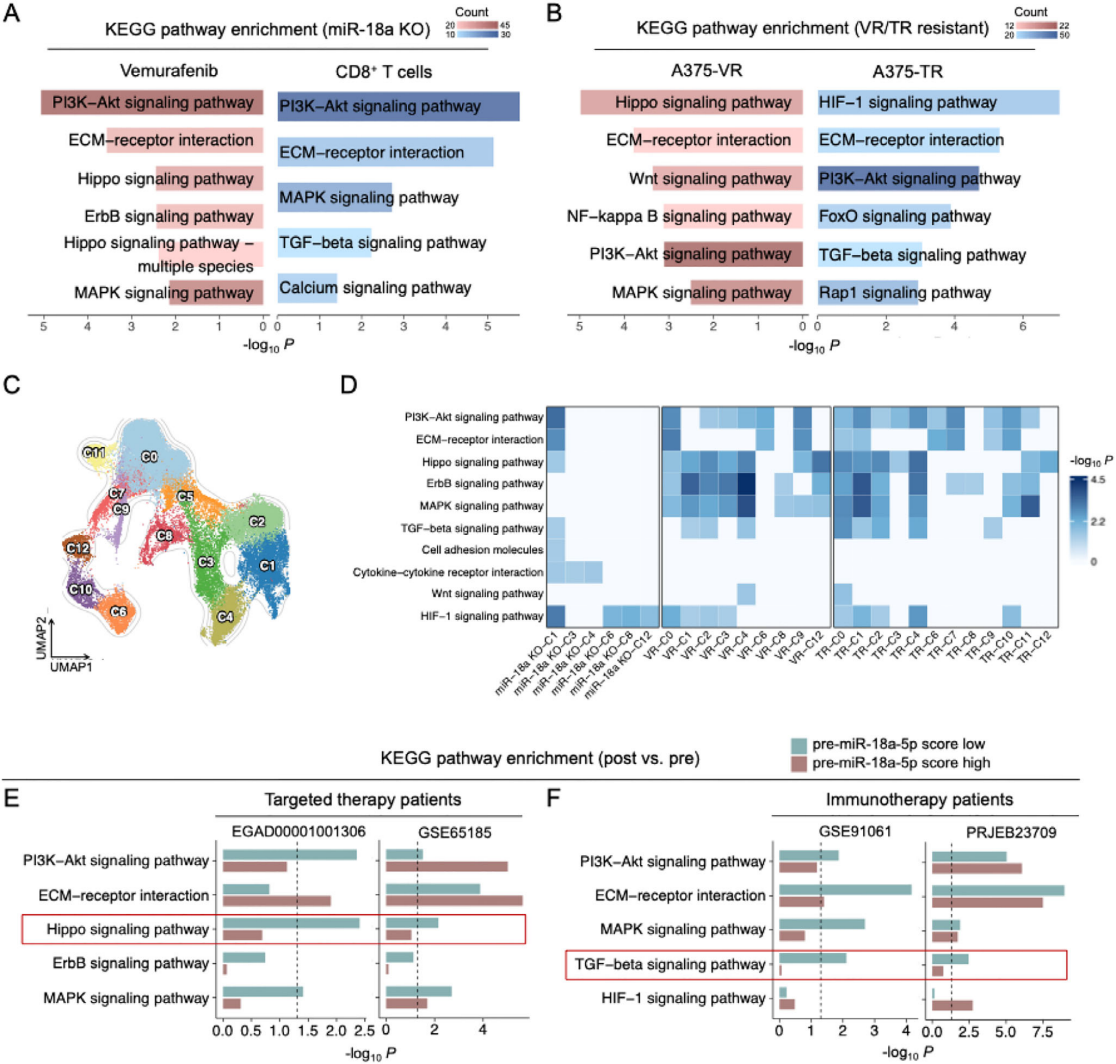
Loss of miR‐18a specifically modulates Hippo and TGF‐β signaling pathways. (A) KEGG pathway enrichment analysis reveals distinct patterns of differentially expressed genes (DEGs) in miR‐18a KO cells compared to control (A375‐Cas9) cells. Left: changes under VEM treatment; right: alterations during CD8^+^ T cell co‐culture. (B) KEGG pathway enrichment analysis revealing distinct patterns of DEGs in A375‐VR cells (left) and A375‐TR cells (right), both compared to WT A375 cells. (C) UMAP visualization of single‐cell transcriptomes from Control, miR‐18a‐KO, A375‐VR, and A375‐TR cells, showing 13 transcriptional clusters (C0–C12). (D) KEGG pathway enrichment of differentially expressed genes in each cluster from miR‐18a‐KO, A375‐VR, and A375‐TR cells compared with the corresponding Control cluster; color intensity represents enrichment significance (−log_10_ *P*). (E) KEGG pathway enrichment analysis of genes differentially expressed post‐treatment versus pre‐treatment in targeted therapy cohorts, stratified by pre‐miR‐18a‐5p score (low versus high). (F) KEGG pathway enrichment analysis of genes differentially expressed post‐treatment versus pre‐treatment in immunotherapy cohorts, stratified by pre‐miR‐18a‐5p score (low versus high).

To explore potential mechanisms by which miR‐18a influences cellular response to targeted therapy and T cell‐mediated immunity in melanoma, we first examined changes in canonical oncogenic signaling pathways, specifically MAPK/ERK and PI3K–AKT, in miR‐18a KO versus A375‐Cas9 control melanoma cells. Treatment of miR‐18a KO cells with VEM, either alone or in combination with trametinib, induced pronounced reactivation of both MAPK and PI3K–AKT pathways, as evidenced by elevated phosphorylation levels of MEK, ERK, and AKT (Figure S9A,B). In contrast, co‐cultures with CD8^+^ T cells revealed no discernible trends in MAPK/ERK signaling reactivation following miR‐18a KO (Figure S9C), suggesting that potentially independent mechanisms may underlie miR‐18a‐mediated modulation of immune resistance. Similar outcomes were observed in miR‐18a KO SK‐MEL‐28 cells (Figure S9D–F). To further characterize the biological mechanisms underlying these resistance effects, we performed Kyoto Encyclopedia of Genes and Genomes (KEGG) pathway enrichment analysis of differentially expressed genes (DEGs) in both control and miR‐18a KO A375 cells under various treatment conditions. This analysis identified significant enrichment of classical BRAFi/MEKi resistance pathways, including the PI3K–AKT, extracellular matrix (ECM)‐receptor interaction, Hippo, ErbB, and MAPK signaling pathways in VEM‐treated cells (Figure 3A and Table S6). Additionally, pathways involved in PI3K–AKT, ECM‐receptor interaction, MAPK, TGF‐β, and calcium signaling were prominently enriched in CD8^+^ T cell co‐cultures (Figure 3A and Table S7).

To validate the involvement of miR‐18a‐associated candidate resistance pathways across diverse treatment regimens and clinical samples, we conducted pathway enrichment analysis on DEGs in A375‐VR cells and A375‐TR cells compared to control A375 cells. Consistent with the enrichment patterns observed in miR‐18a KO cells, this analysis revealed significant enrichment of signaling pathways, including Hippo, ECM‐receptor interaction, Wnt, NF‐κB, PI3K–AKT, and MAPK signaling pathways in A375‐VR (Figure 3B and Figure S9G and Table S20), while A375‐TR exhibited enrichment in HIF‐1, ECM‐receptor interaction, PI3K–AKT, FoxO, TGF‐β, and Rap1 signaling pathways (Figure 3B and Figure S9H and Table S21).

To obtain a unified view of miR‐18a‐related transcriptional programs, we first performed an integrated analysis of scRNA‐seq data from miR‐18a KO, A375‐VR, and A375‐TR cells together with control A375 cells. Quality control metrics for all four conditions are shown in Figures S10A–C. Joint clustering identified 13 transcriptionally distinct melanoma cell states (Figure 3C and Figure S10D). Each of the three perturbed conditions (miR‐18a KO, VR, and TR) exhibited substantial numbers of DEGs relative to control cells, with VR and TR cells showing a larger DEG burden than miR‐18a KO cells (Figure S10E). Notably, most of the key pathways enriched in bulk transcriptomic analyses, including PI3K–AKT, ECM–receptor interaction, Hippo, and TGF‐β signaling, were also observed at the single‐cell level in specific clusters (Figure 3D).

To further investigate how these pathways relate to clinical responses under different therapeutic contexts, we next examined pathway activity in the collected patient cohorts receiving targeted therapy or immunotherapy. Patients were stratified into low and high groups according to their pre‐miR‐18a‐5p score. In targeted therapy cohorts, Hippo signaling showed preferential enrichment in patients with high pre‐miR‐18a‐5p scores, whereas in immunotherapy cohorts, TGF‐β signaling was specifically enhanced in the high pre‐miR‐18a‐5p group (Figure 3E,F). Together, these data suggest an association between miR‐18a and the activation of Hippo and TGF‐β signaling, raising the possibility that miR‐18a‐associated resistance may, at least in part, be driven by the activation of these pathways.

### 2.4 miR‐18a Deficiency Confers VEM Resistance Through the AJUBA‐Mediated Hippo Signaling Pathway

**FIGURE 4:**
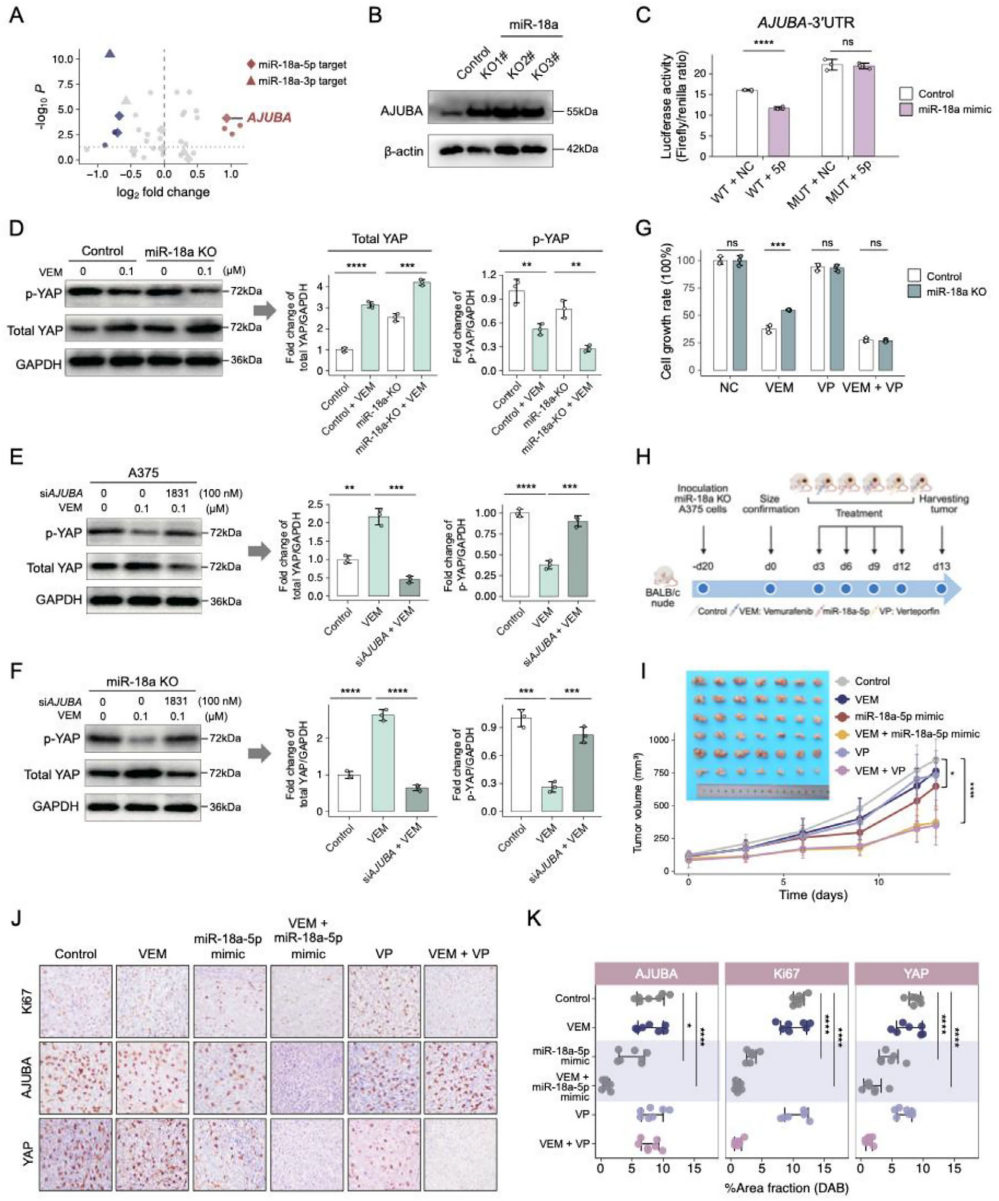
miR‐18a regulates the Hippo signaling pathway in VEM resistance through targeting *AJUBA*. (A) Volcano plot showing differential expression of Hippo pathway genes, with miR‐18a‐5p targets (diamonds) and miR‐18a‐3p targets (triangles) highlighted and *AJUBA* labeled. (B) Western blot of AJUBA protein levels in A375‐Cas9 (control) and miR‐18a KO A375 cells. (C) Dual‐luciferase reporter assay demonstrating that miR‐18a‐5p mimics suppress wild‐type (WT) *AJUBA* 3′UTR activity but not mutant (MUT) 3′UTR. (D) Western blot analysis of YAP activity in A375‐Cas9 (control) and miR‐18a KO A375 cells treated with or without VEM (0.1 µm). (E, F) Western blot analysis of YAP activity changes in A375‐Cas9 (E) and miR‐18a KO A375 (F) cells following *AJUBA* knockdown by siRNA (1831, 100 nm). (G) Cell proliferation of A375‐Cas9 (control) and miR‐18a KO cells under vehicle control (NC), 0.1 µm VEM, 0.2 µm verteporfin (VP), or combination (0.1 µm VEM + 0.2 µm VP) conditions. (H) Schematic of the experimental design for miR‐18a KO A375 xenograft studies in BALB/c nude mice. Mice received injections of vehicle control, VEM (75 mg/kg), VP (50 mg/kg), miR‐18a‐5p mimic (2 OD), or their combinations. (I) Representative tumor images and tumor growth curves in xenograft mice under various treatments. (J) Representative immunohistochemical (IHC) images of AJUBA and YAP in tumor tissues. K) Quantitative analysis of AJUBA and YAP IHC staining intensities. Data represent means ± SD; *n* = 3 biologically independent samples for panels C–G, and n = 7 for panels I and K. Two‐way ANOVA followed by Tukey's post hoc test is used to calculate *P*‐values in panel I and unpaired two‐tailed Student's *t*‐test is used to calculate *P*‐values in panels C–G, and K: *, *P* < 0.05; **, *P* < 0.01; ***, *P* < 0.001; ****, *P* < 0.0001; ns, not significant.

Accumulating evidence has suggested an association between Hippo signaling dysregulation and resistance to BRAFi/MEKi in melanoma [31, 32]. However, the upstream molecular regulators governing these resistance mechanisms and their precise modes of action remain poorly understood. Through integrative analysis of functional screening data, cellular models, and clinical datasets, we identified miR‐18a, particularly its mature form miR‐18a‐5p, as a critical mediator of BRAFi/MEKi treatment resistance linked to Hippo pathway activation. Our systematic investigations of Hippo pathway components revealed that *AJUBA* expression is markedly upregulated following miR‐18a KO, and bioinformatic analysis using TargetScan identified *AJUBA* as a high‐confidence target of miR‐18a‐5p (Figure 4A and Figure S11A and Tables S6 and S8). These findings suggest that *AJUBA* serves as a pivotal downstream effector through which miR‐18a‐5p modulates drug resistance. To validate this regulatory relationship, we performed RT‐qPCR and Western blot analyses on miR‐18a KO clones, confirming elevated *AJUBA* expression at both mRNA and protein levels (Figure 4B and Figure S11B). To further assess whether *AJUBA* is a direct target of miR‐18a‐5p, we employed a dual‐luciferase reporter assay. The results supported that a conserved sequence within the *AJUBA* 3′UTR is specifically recognized and substantially repressed by miR‐18a‐5p in vitro (Figure 4C).

As a negative regulatory cofactor of the Hippo pathway, AJUBA facilitates YAP/TAZ activation by inhibiting the kinase activity of LATS1/2 [33, 34], thereby promoting nuclear accumulation of YAP/TAZ and transcription of pro‐survival genes that drive BRAFi/MEKi resistance in melanoma. To determine whether miR‐18a‐5p mediates resistance through *AJUBA*‐dependent regulation of downstream Hippo signaling components, we examined YAP expression and phosphorylation status under various experimental conditions in A375 cells. Consistent with our hypothesis, miR‐18a KO resulted in increased total YAP levels and decreased phosphorylated YAP (p‐YAP), indicative of enhanced YAP activity. Notably, VEM treatment further amplified YAP accumulation while suppressing p‐YAP, with these effects being most pronounced in miR‐18a KO cells (Figure 4D). Furthermore, siRNA‐mediated knockdown of *AJUBA* in A375 and miR‐18a KO A375 cells significantly attenuated total YAP upon VEM treatment (Figure 4E,F and Figure S11C). Collectively, these findings support that miR‐18a loss relieves the post‐transcriptional suppression of *AJUBA*, thereby promoting YAP expression and activity. VEM treatment further potentiated YAP accumulation in miR‐18a‐deficient cells, ultimately contributing to melanoma drug resistance.

To comprehensively characterize the drug‐resistant phenotype and explore potential therapeutic strategies to overcome resistance, we evaluated verteporfin, a photosensitizer clinically approved for treating pathological ocular neovascularization that also functions as a YAP inhibitor by disrupting YAP‐TEAD interactions [35]. Cell proliferation assays revealed that verteporfin treatment, either as monotherapy or in combination with VEM, significantly mitigated resistance and re‐sensitized miR‐18a KO A375 cells to VEM (Figure 4G). We further evaluated VT3989 [36], another YAP inhibitor that disrupts YAP‐TEAD interactions, and obtained comparable results (Figure S11D). AJUBA overexpression significantly enhanced VEM resistance (Figure S11E,F) and upregulated expression of the YAP pathway downstream target genes *CTGF* and *CYR61* (Figure S11G). Conversely, *AJUBA* knockdown markedly suppressed *CTGF* and *CYR61* expression (Figure S11H), further underscoring the critical role of AJUBA in mediating melanoma VEM resistance through YAP pathway activation. We further validated these findings in vivo using BALB/c nude mice bearing subcutaneous xenografts derived from miR‐18a KO A375 cells. After establishing tumors of uniform size (day 20 post‐injection), mice were subjected to multiple therapeutic interventions, including intratumoral delivery of miR‐18a‐5p mimics using JetPEI (Polyplus‐Transfection) and systemic administration of VEM or verteporfin, either alone or in combination (Figure 4H). Intratumoral miR‐18a‐5p mimic injection substantially reduced xenograft volume and weight compared to vehicle‐treated controls, whereas single‐agent VEM or verteporfin exhibited only modest tumor suppression effects (Figure 4I and Figure S11I). Importantly, combination therapy with VEM plus miR‐18a‐5p mimics or VEM plus verteporfin markedly suppressed tumor growth rates in xenograft‐bearing mice, suggesting that effective YAP inhibition can overcome miR‐18a loss‐mediated VEM resistance (Figure 4I). Immunohistochemical (IHC) analysis of proliferation markers (Ki67) and Hippo pathway components (AJUBA, YAP) showed that combination treatments effectively suppressed tumor proliferation and partially reversed Hippo pathway dysregulation in vivo (Figure 4J,K). Notably, monotherapy with miR‐18a‐5p mimics also modestly reduced proliferation and expression of downstream Hippo signaling targets in vivo (Figure 4I–K). To further investigate these effects, we employed LoQANT [37] to quantify the nuclear‐to‐cytoplasmic intensity ratio of YAP. Consistent with our in vitro observations, the in vivo findings revealed that miR‐18a‐5p mimic overexpression significantly reduced YAP's nuclear‐to‐cytoplasmic intensity ratio in both control and VEM‐treated conditions (Figure S11J). These results demonstrate that miR‐18a‐5p suppresses YAP nuclear translocation and activation regardless of VEM treatment status. Taken together, our data support the conclusion that miR‐18a deficiency, particularly loss of miR‐18a‐5p, confers VEM resistance in melanoma through derepression of the AJUBA‐YAP axis and consequent Hippo pathway dysregulation.

### 2.5 miR‐18a Deficiency Impairs CD8^+^ T Cell Cytotoxicity Through a THBS1–CD47‐Dependent Mechanism

**FIGURE 5:**
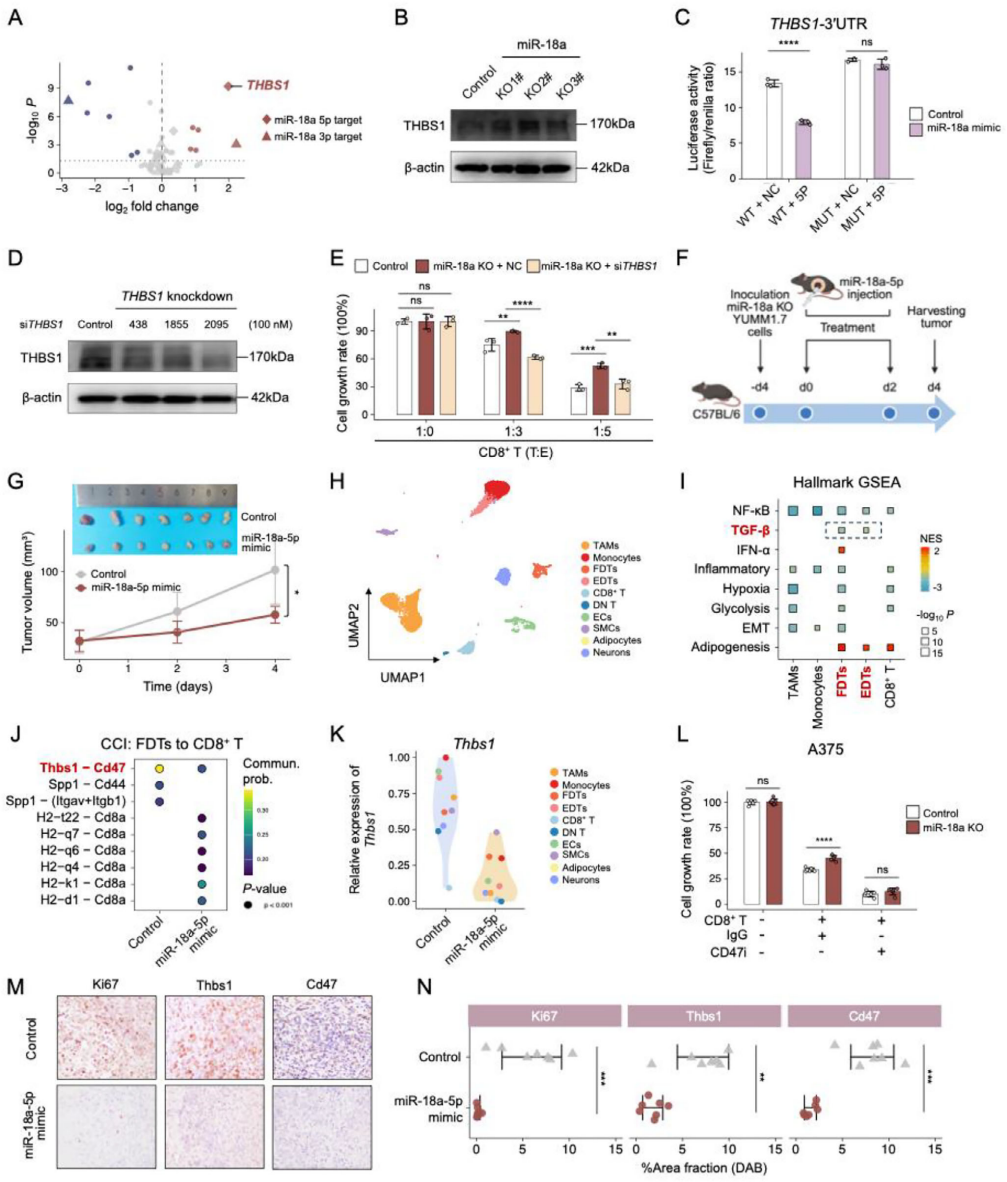
miR‐18a regulates the TGF‐β signaling pathway to confer CD8^+^ T cell tolerance by targeting *THBS1*. (A) Volcano plot showing differential expression of TGF‐β pathway genes, with miR‐18a‐5p targets (diamonds) and miR‐18a‐3p targets (triangles) highlighted and *THBS1* labeled. (B) Western blot of THBS1 in A375‐Cas9 (control) and miR‐18a KO cells. (C) Dual‐luciferase reporter assay showing miR‐18a‐5p mimics suppress WT *THBS1* 3′UTR activity but not MUT 3′UTR. NC: scrambled mimic control. (D) Western blot confirming siRNA‐mediated *THBS1* knockdown (438, 1955, and 2095) in miR‐18a KO A375 cells. Control: scrambled RNA. (E) Cell proliferation of A375‐Cas9 (control) and miR‐18a KO cells co‐cultured with CD8^+^ T cells at various T:E ratios with scrambled RNA (NC) or si*THBS1* (2095). (F) Schematic of C57BL/6 murine tumor model with miR‐18a‐5p mimic treatment. Mice were inoculated with miR‐18a KO YUMM1.7 cells and subsequently injected with 20 µL of scrambled mimic control or miR‐18a‐5p mimics. (G) Representative tumor images and tumor growth curves in miR‐18a‐5p mimic‐treated versus control mice. (H) Single‐cell UMAP of tumor tissues from control and miR‐18a‐5p mimic‐treated mice. (I) Hallmark pathway enrichment of DEGs (miR‐18a‐5p mimic versus control), showing TGF‐β suppression. FDTs: Fibroblast‐Derived Tumor cells; EDTs: Epithelial‐Derived Tumor cells. (J) Signaling interactions between FDTs and CD8^+^ T cells in control versus miR‐18a‐5p mimic‐treated mice. (K) *Thbs1* expression across cell types in control and miR‐18a‐5p mimic‐treated mice. (L) Cell proliferation of A375‐Cas9 (control) and miR‐18a KO A375 cells with/without CD8^+^ T cell and CD47 inhibitor (magrolimab; isotype IgG control). (M, N) Representative IHC images (M) and quantification (N) of Ki67, Cd47, and Thbs1 in murine tumor tissues. Data represent means ± SD; *n* = 3 biologically independent samples for panels C and E, *n* = 6 for panel L, and *n* = 7 for panels G and N. Two‐way ANOVA followed by Tukey's post hoc test is used to calculate *P*‐values in panel G and unpaired two‐tailed Student's *t*‐test is used to calculate *P*‐values in panels C, E, L, and N: *, *P* < 0.05; **, *P* < 0.01; ***, *P* < 0.001; ****, *P* < 0.0001; ns, not significant.

Analysis of multi‐stage samples from both T cell co‐culture models and clinical immunotherapy cohorts suggested that the loss of miR‐18a in melanoma is associated with impaired CD8^+^ T cell cytotoxic capability, potentially involving TGF‐β signaling pathways (Figure 3A,B). TGF‐β, in concert with the tumor microenvironment, promotes tumor heterogeneity and immune suppression across various cancers and can modulate drug resistance [38, 39, 40]. To investigate the underlying mechanisms in melanoma, we first examined potential direct miR‐18a‐5p target genes within the TGF‐β pathway and observed marked upregulation of thrombospondin‐1 (*THBS1*), which encodes a key regulatory protein frequently reported to modulate or be induced through TGF‐β and ECM‐related signaling pathways that shape immune suppression [41, 42, 43] (Figure 5A and Figure S12A and Tables S7 and S8). These observations, together with previous literature, suggest that *THBS1* may be an important candidate molecule through which miR‐18a‐5p modulates the tumor microenvironment, thereby contributing to resistance to T cell‐mediated cytotoxicity. In miR‐18a KO clones, RT‐qPCR and Western blot assays showed that the absence of miR‐18a led to increased *THBS1* expression, consistent with negative regulation by miR‐18a (Figure 5B and Figure S12B). Dual‐luciferase reporter assays demonstrated that a sequence in the *THBS1* 3′UTR was recognized by miR‐18a‐5p and significantly suppressed reporter gene expression in vitro (Figure 5C). Furthermore, siRNA‐mediated knockdown of *THBS1* in miR‐18a KO A375 cells significantly attenuated resistance to CD8^+^ T cell‐mediated killing across varying T:E ratios (Figure 5D,E). These findings support a functional role for the miR‐18a–THBS1 axis in attenuating CD8^+^ T cell cytotoxicity and shaping an immunosuppressive tumor microenvironment in melanoma.

To investigate the role of miR‐18a in shaping the immune microenvironment in melanoma in vivo, we generated miR‐18a KO monoclonal cell lines using YUMM1.7, a murine BRAF V600E melanoma cell line [44] (Figure S12C,D). We performed short‐term (4‐day) subcutaneous engraftment of miR‐18a KO YUMM1.7 cells in immunocompetent C57BL/6 mice and selected mice with consistent tumor sizes for two rounds of intratumoral injection with miR‐18a‐5p mimic/JetPEI complexes (Figure 5F). Initially, miR‐18a KO YUMM1.7 grafts grew progressively under the influence of the host immune system. As expected, treatment with miR‐18a‐5p mimic significantly attenuated increases in tumor volume and weight compared to control‐treated miR‐18a KO tumor‐bearing mice (Figure 5G and Figure S12E), indicating that restoration of miR‐18a‐5p facilitates more rapid immune‐mediated killing of melanoma cells.

To better understand how miR‐18a‐5p reshapes the melanoma immune microenvironment, we performed scRNA‐seq on harvested engrafted tumors with and without miR‐18a‐5p mimic treatment. Analysis of single‐cell transcriptomes from engrafted tumors and tumor‐infiltrating immune cells identified multiple clusters, including fibroblast‐derived tumor cells (FDTs), epithelial‐derived tumor cells (EDTs), CD8^+^ T cells, and tumor‐associated macrophages (TAMs) (Figure 5H and Figure S12F–I). Compared to control‐treated miR‐18a KO YUMM1.7‐grafted mice, those receiving miR‐18a‐5p mimic exhibited significant differential gene expression across various cell types, with the exception of EDTs (Figure S12J). Notably, GSEA suggested reduced enrichment of TGF‐β signaling in FDTs and EDTs following miR‐18a‐5p mimic treatment (Figure 5I). Concurrently, CellChat analysis suggested reduced inferred Thbs1‐related communication between FDTs/EDTs and CD8^+^ T cells, including lower inferred Thbs1–Cd47 interaction probability in the miR‐18a‐5p mimic group (Figure 5J and Figure S12K). Additionally, *Thbs1* expression was significantly downregulated across multiple cell types following miR‐18a‐5p mimic treatment (Figure 5K), supporting the hypothesis that melanoma‐derived ECM protein Thbs1 likely mediates CD8^+^ T cell exhaustion or impedes sustained T cell activation through interaction with Cd47 on CD8^+^ T cells [42, 45].

To corroborate our single‐cell observations, CD47 was blocked using magrolimab in activated CD8^+^ T cells co‐cultured with miR‐18a KO A375 cells, which resulted in rapid and effective tumor cell clearance (Figure 5L and Figure S12L). IHC staining for Ki67, Thbs1, and CD47 in harvested engrafted tumors revealed that miR‐18a‐5p mimic treatment significantly reduced tumor proliferation and the levels of Thbs1 and Cd47 in vivo (Figure 5M,N). Using multiplex immunofluorescence staining, we further confirmed diminished colocalization of Thbs1 and Cd47 at tumor‐infiltrating CD8^+^ T cell interfaces following miR‐18a‐5p mimic treatment (Figure S12M). Functionally, CD107a degranulation assays [46, 47] demonstrated that miR‐18a KO significantly impaired the cytotoxic capacity of activated CD8^+^ T cells against A375 cells. Importantly, blocking the THBS1‐CD47 axis with magrolimab markedly restored CD8^+^ T cell cytotoxicity against miR‐18a‐KO A375 cells (Figure S12N), supporting a functional contribution of the THBS1–CD47 axis to the immunosuppressive phenotype induced by miR‐18a loss. Consistent with these in vitro findings, Cd47 blockade in vivo significantly reduced both tumor volume and weight (Figure S12O–R), further supporting an important role for THBS1‐CD47 signaling in melanoma immune evasion. Collectively, our experiments support a mechanism by which miR‐18a modulates CD8^+^ T cell cytotoxicity through regulation of THBS1 production and its interaction with CD47. These findings highlight a potential therapeutic strategy targeting the THBS1‐CD47 signaling axis to overcome the immunosuppressive conditions associated with melanoma resistance to immunotherapy.

### 2.6 hnRNP A1 is an Upstream Regulator of miR‐18a‐Mediated Pleiotropic Resistance

**FIGURE 6:**
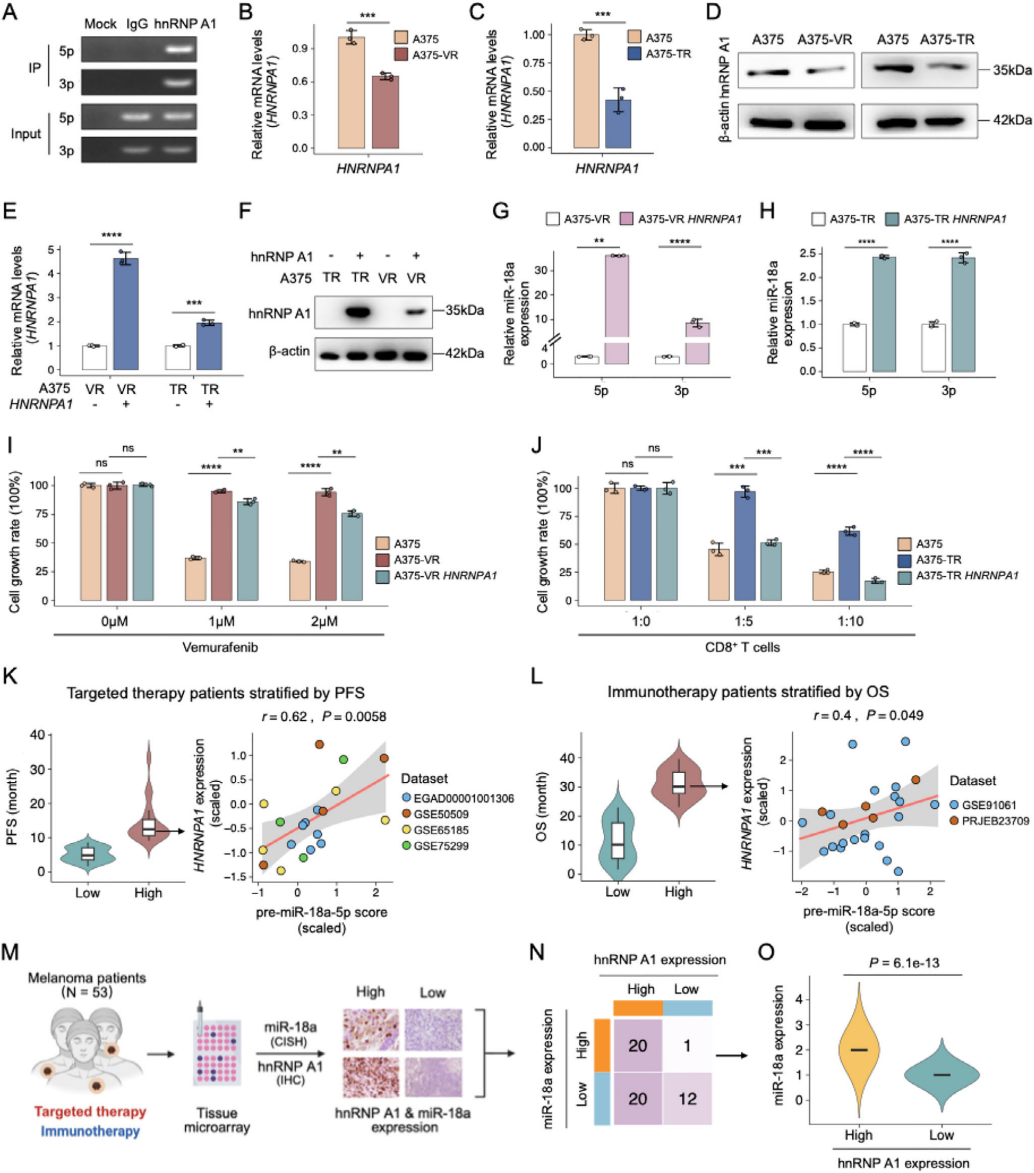
hnRNP A1 regulation of miR‐18a in melanoma models and clinical cohorts. (A) RNA immunoprecipitation (RIP) assay using anti‐hnRNP A1 or isotype IgG control antibodies, followed by RT‐PCR detection of miR‐18a isoforms. Nuclease‐free water served as the mock control. (B, C) Relative *HNRNPA1* mRNA levels in A375‐VR (B) and A375‐TR (C) versus A375 (control) cells. (D) Western blot of hnRNP A1 protein in A375 (control), A375‐VR, and A375‐TR cells. (E, F) *HNRNPA1* mRNA (E) and protein (F) levels in A375‐VR and A375‐TR cells with or without *HNRNPA1* overexpression; protein levels were determined by Western blot using an anti‐FLAG antibody. (G, H) miR‐18a‐5p/3p expression in A375‐VR (G) and A375‐TR (H) cells upon *HNRNPA1* overexpression. (I, J) Cell viability of A375 (control), A375‐VR/TR, and hnRNP A1‐rescued cells under increasing VEM concentrations (I) or CD8^+^ T cell co‐culture at various T:E ratios (J). (K, L) Association between *HNRNPA1* expression and pre‐miR‐18a‐5p score in targeted therapy (K) and immunotherapy (L) cohorts. Left: patients stratified into high and low groups based on progression‐free survival (PFS, K) or overall survival (OS, L). Right: correlation between pre‐miR‐18a‐5p score (scaled) and *HNRNPA1* expression in the survival‐high group. (M) Schematic of an independent validation cohort assessing hnRNP A1 and miR‐18a levels in patients receiving targeted therapy or immunotherapy. (N) Contingency heatmap of hnRNP A1 and miR‐18a expression in melanoma tissues from the validation cohort, categorized by high/low expression. (O) miR‐18a expression distribution in melanoma tissues stratified by high/low hnRNP A1 expression. Data represent means ± SD; *n* = 3 biologically independent samples for panels B, C, E, and G–J. Unpaired two‐tailed Student's *t*‐test is used to calculate *P*‐values in panels B, C, E, and G–J: **, *P* < 0.01; ***, *P* < 0.001; ****, *P* < 0.0001; ns, not significant.

Analysis of archival melanoma samples revealed that the miR‐17‐92 cluster, encoded by *MIR17HG*, typically lacks mutations and exhibits no significant copy number changes (Figure S4A,B). This suggests that dysregulation of miR‐18a during melanoma progression and treatment may be governed by non‐genetic factors associated with the miR‐18a host gene. Previous studies have identified the multifunctional RNA‐binding protein hnRNP A1 as critical for the processing and stability of miR‐18a precursor RNA [48, 49]. We therefore hypothesized that hnRNP A1 might underlie the miR‐18a‐mediated cross‐resistance observed in both targeted therapies and T cell‐mediated cytotoxicity in melanoma. Initial RNA immunoprecipitation (RIP) assays in A375 melanoma cells confirmed the binding of hnRNP A1 to miR‐18a (Figure 6A). Concurrent RT‐qPCR and Western blot analyses demonstrated that, compared to parental A375 cells, *HNRNPA1* mRNA and protein levels were downregulated in both A375‐VR and A375‐TR cell lines (Figure 6B–D). This observation implicates hnRNP A1 as a potential upstream regulator of miR‐18a‐driven dual resistance in melanoma. To validate this regulatory relationship, we overexpressed hnRNP A1 in the A375‐VR and A375‐TR melanoma cell lines, which resulted in marked upregulation of miR‐18a levels (Figure 6E–H). Importantly, hnRNP A1 overexpression significantly sensitized resistant cells to VEM treatment and enhanced their susceptibility to CD8^+^ T cell‐mediated cytotoxicity (Figure 6I,J), demonstrating that hnRNP A1 modulates treatment resistance mechanisms. Conversely, *HNRNPA1* KO in A375‐VR/TR cells substantially diminished miR‐18a expression and conferred increased resistance to both VEM treatment and CD8^+^ T cell‐mediated killing, while hnRNP A1 re‐expression reversed this resistant/immune‐evasive phenotype (Figure S13). Mechanistically, treatment with VEM and activated CD8^+^ T cells decreased *HNRNPA1* mRNA and protein levels, accompanied by significantly reduced miR‐18a expression (Figure S14). To establish the directionality of this regulatory axis, we examined whether miR‐18a loss reciprocally affected *HNRNPA1* expression. Notably, miR‐18a depletion did not alter *HNRNPA1* mRNA or protein levels, regardless of VEM and activated CD8^+^ T cell treatment (Figure S15), confirming that hnRNP A1 functions upstream of miR‐18a. Collectively, these findings establish a regulatory cascade whereby VEM and activated CD8^+^ T cell treatment reduces *HNRNPA1* expression, leading to decreased miR‐18a levels that subsequently promote drug resistance and immune evasion.

Further analysis of transcriptomic data from 61 melanoma patients receiving targeted therapy revealed that, within the PFS‐high group, *HNRNPA1* expression was positively correlated with pre‐miR‐18a‐5p scores (*r* = 0.62, *P* = 0.0058; Figure 6K). Similarly, in 59 melanoma patients undergoing immunotherapy, *HNRNPA1* expression levels in the OS‐high group were positively associated with pre‐miR‐18a‐5p scores (*r* = 0.4, *P* = 0.049; Figure 6L). To further validate the relationship between hnRNP A1 and miR‐18a expression levels in melanoma patients receiving targeted therapy or immunotherapy, we retrospectively collected formalin‐fixed paraffin‐embedded (FFPE) surgical tissue specimens and clinical outcome data from melanoma patients treated at Tianjin Medical University Cancer Hospital between 2019 and 2023. We analyzed 53 patient samples from individuals treated with BRAFi/MEKi or ICIs, assessing hnRNP A1 protein levels and miR‐18a expression via tissue microarray‐based IHC and chromogenic in situ hybridization (CISH), respectively (Figure 6M and Table S22). Patient samples were stratified into groups based on hnRNP A1 expression levels determined by IHC. We observed that higher hnRNP A1 expression was associated with elevated miR‐18a levels (*P* = 6.1 × 10^−13^) in patients receiving both targeted and immune therapies (Figure 6M–O and Figure S16 and Table S23). These findings collectively demonstrate that hnRNP A1 plays an indispensable role in regulating miR‐18a and its associated resistance pathways in melanoma, highlighting its potential as a valuable biomarker of therapeutic response in *BRAF*‐mutant melanoma and as a candidate therapeutic target for overcoming treatment resistance.

## 3 Discussion

Cross‐resistance between targeted therapy and immune evasion remains a significant challenge in cancer treatment. Through unbiased CRISPR KO screening, we identified miRNAs, particularly miR‐18a, as pivotal modulators of pleiotropic resistance mechanisms in melanoma. Our multi‐omic analysis demonstrated that miR‐18a orchestrates resistance pathways influencing both drug response and immune surveillance across cellular models and clinical samples. By dissecting the downstream effects of miR‐18a loss, we uncovered MAPK/ERK pathway reactivation as a key intracellular mechanism and further identified novel resistance niches within the tumor microenvironment. Mechanistic studies elucidated how the Hippo and TGF‐β pathways modulate melanoma resistance, addressing longstanding ambiguities regarding Hippo pathway dysfunction in BRAFi/MEKi resistance and unveiling new T cell immune regulation mechanisms. Notably, miR‐18a reintroduction reversed resistance phenotypes in vivo, underscoring its therapeutic potential [50].

Numerous studies have explored CRISPR‐based functional screenings targeting miRNAs in tumor cell models [17, 51, 52, 53, 54, 55]. Some employed the GeCKO library [18], which combines protein‐coding and miRNA genes, potentially diluting screening power. Others designed in‐house sgRNAs specifically targeting genome‐wide microRNAs, though the coverage and scope of readouts could be extended. By redesigning a more comprehensive microRNA KO library and applying it to multiple cellular phenotypes, we fortuitously discovered the dual role of miR‐18a in mediating resistance to BRAFi and T cell cytotoxicity in melanoma. Unlike traditional gene‐level KO screens [56], miRNA‐level screens exhibit moderate power and effect, likely due to the generally mild biological effects of most miRNAs. Moreover, CRISPR‐mediated non‐homologous end joining KO does not precisely control mature miRNA expression and may introduce heterogeneity and off‐target effects. Therefore, employing endogenous RNA editing systems such as CRISPR‐Cas13d to perturb miRNA precursors or mature miRNAs represents a promising future direction [57, 58, 59, 60].

miR‐18a, closely associated with tumorigenesis, exhibits oncogenic or tumor‐suppressive properties depending on the cancer context [23, 24]. However, research on miR‐18a has often been confined to static conditions, neglecting the dynamic interplay between its multi‐target regulatory network and tumor capabilities. Through CRISPR screening, we systematically delineated the role of miR‐18a‐5p in BRAF V600E mutant melanomas during responses to BRAFi/MEKi treatments and T cell immune cytotoxicity, shedding light on acquired tolerance mechanisms and the previously uncharacterized common upstream mechanisms in melanoma cross‐resistance. Investigating miR‐18a‐mediated pleiotropic resistance in non‐BRAF V600E mutant melanomas and other tumor types warrants further exploration.

We identified that miR‐18a loss mediates resistance in melanoma through well‐established pathways, including the Hippo pathway via YAP [31, 32] and T cell cytotoxic resistance through TGF‐β signaling‐related cellular communication [38]. Although the independence of these pathways warrants further clinical investigation, previous research has demonstrated that Hippo pathway defects can drive YAP‐induced PD‐L1 expression, leading to immune evasion in BRAFi‐resistant melanoma [9], with *THBS1* mediating YAP‐induced cell invasion [61]. These findings suggest that Hippo pathway dysfunction may couple with T cell immune tolerance as early drivers of resistance. Furthermore, the literature supports that these two pathways exhibit bidirectional crosstalk in certain contexts (e.g., YAP acting as a TGF‐β/Smad co‐activator [62]; YAP‐induced TGF‐β/SMAD7 feedback [63]). Definitively dissecting the causal relationship and mutual dependence between Hippo and TGF‐β signaling in the context of miR‐18a‐induced melanoma resistance would require additional experiments in future work. Notably, clinical practice indicates that ipilimumab‐containing regimens are no longer recommended for BRAF‐mutated patients progressing after BRAFi/MEKi treatment [5, 64], with numerous cases showing subsequent failure to respond to combined immuno‐ and targeted therapies [65]. Through scRNA‐seq analysis of miR‐18a KO monoclonal cells alongside A375‐VR and A375‐TR models, we uncovered that specific cellular subpopulations manifest distinct resistance pathways, emphasizing the heterogeneity within resistant melanoma cells.

Our study also preliminarily explored upstream mechanisms of miR‐18a dysfunction. Although current melanoma cohorts lack significant *MIR17HG* mutations, this may be attributable to limited sample sizes and the absence of longitudinal genomic characterizations that track clonal evolution during therapy [66]. Additionally, epigenetic remodeling may alter *MIR17HG* expression or miR‐18a processing. Our investigation of hnRNP A1, a molecule specifically regulating miR‐18a, revealed coordinated changes during melanoma responses to targeted therapy and T cell cytotoxicity. Preliminary results indicate that miR‐18a knockout does not affect hnRNP A1 expression. However, whether hnRNP A1 correlates with miR‐18a‐mediated cross‐resistance or represents a feedback mechanism requires further investigation.

Regarding our CRISPR screen methodology, CD8^+^ T cells were isolated from three independent healthy donors and pooled. We acknowledge that inter‐donor variability in cytotoxic activity represents an inherent limitation of primary human T cell models. However, we posit that this variability may enhance translational relevance by capturing the heterogeneity of T cell responses observed in clinical settings. Critically, genes conferring resistance across diverse T cell phenotypes are more likely to represent broadly applicable therapeutic targets rather than donor‐specific phenomena. The current study prioritized establishing the screening platform and identifying candidate mechanisms. In subsequent studies, we will utilize tumor‐reactive T cells derived from patients and antigen‐specific killing assays to validate these findings and further confirm the therapeutic potential of these pathways.

This study establishes a multi‐layered mechanistic framework for miR‐18a‐mediated pleiotropic resistance in melanoma, with findings stratified by evidence type. Conclusions supported by direct experimental evidence include: CRISPR‐mediated miR‐18a knockout confers stable resistance to VEM and CD8^+^ T cell cytotoxicity across multiple melanoma cell lines, with phenotypes rescued by miR‐18a mimic restoration; *AJUBA* and *THBS1* are validated as direct miR‐18a‐5p targets via luciferase reporter assays, with their functional roles in drug resistance and immune evasion confirmed through siRNA knockdown, pharmacological inhibition, and in vivo mouse models; and hnRNP A1 is established as an upstream regulator of miR‐18a through RIP assays, directional rescue experiments, and clinical tissue validation. In contrast, conclusions derived from computational inference—including ssGSEA‐based miR‐18a activity scores and their associations with clinical outcomes, pathway enrichment in patient cohorts, and CellChat‐inferred intercellular communication—should be regarded as hypothesis‐generating. These observations are constrained by limited sample sizes, indirect miRNA quantification, and inconsistent statistical robustness after covariate adjustment, and therefore require prospective validation in larger, well‐annotated cohorts before causal conclusions can be drawn.

In conclusion, our research reveals the critical role of miR‐18a in orchestrating pleiotropic resistance mechanisms across multiple temporal phases and resistance contexts in melanoma. This work represents the first systematic, genome‐wide CRISPR screen establishing the causal contribution of miRNAs to melanoma cross‐resistance, extending beyond conventional protein‐coding drivers. By elucidating miR‐18a's regulatory interplay with the Hippo and TGF‐β pathways, we demonstrate that non‐coding RNAs function as central nodes at the intersection of targeted therapy resistance and immune evasion. While our findings advance mechanistic understanding and illuminate therapeutic opportunities for clinical translation, the temporal dynamics of miR‐18a regulation during the evolution from acute to chronic resistance warrant further investigation in longitudinal studies. Moving forward, the development of targeted delivery systems to modulate miR‐18a expression in tumors represents a promising avenue for therapeutic intervention.

## 4 Materials and Methods

### 4.1 Cell Lines

Human melanoma cell line A375 (American Type Culture Collection [ATCC], CRL‐1619) and human embryonic kidney cell line HEK293FT (Thermo Fisher Scientific, R70007) were cultured in Dulbecco's Modified Eagle Medium (DMEM; Thermo Fisher Scientific, 11965092) supplemented with 10% fetal bovine serum (FBS; Thermo Fisher Scientific, 10437028). Human melanoma cell line SK‐MEL‐28 (ATCC, HTB‐72) was maintained in Eagle's Minimum Essential Medium (EMEM; ATCC, 30–2003) supplemented with 10% FBS. Murine melanoma cell line YUMM1.7 (ATCC, CRL‐3362) was cultured in DMEM/F‐12 (Thermo Fisher Scientific, 11320033) supplemented with 10% FBS. All cell lines were maintained at 37°C in a humidified atmosphere containing 5% CO_2_ and routinely tested for mycoplasma contamination (Lonza, LT07‐218). Cells were passaged upon reaching 80%–90% confluence using 0.25% Trypsin‐EDTA (Gibco, 25200‐056) at split ratios of 1:4 for melanoma lines and 1:5 for HEK293FT cells.

### 4.2 Design and Construction of the miRNA CRISPR KO sgRNA Library

A comprehensive, non‐redundant set of 2185 human miRNAs was curated by integrating 1901 entries from miRBase v21 [20] with 367 miRNAs from FANTOM5 [21]. sgRNA design was guided by pre‐miRNA structural prioritization [19], targeting four regions in order of priority: Region A (8‐nucleotide [nt] seed region, positions 1–8), Region B (4‐nt sequences flanking Drosha cleavage sites), Region C (14‐nt sequence adjacent to the seed region), and Region D (remaining regions). Using FlashFry [67], 8598 sgRNAs targeting these miRNAs were generated and supplemented with 1000 non‐targeting control sgRNAs. Oligonucleotides were synthesized by Synbio Technologies and cloned into the LentiGuide‐Puro vector (Addgene, #52963) via Gibson assembly and were then packaged into lentiviral particles. Lentiviral production was performed in HEK293FT cells by co‐transfecting the sgRNA library with the psPAX2 (Addgene, #12260) and pMD2.G (Addgene, #12259) packaging plasmids using Lipofectamine 2000 (Thermo Fisher Scientific, 11668019). Viral supernatants were concentrated by ultracentrifugation (Beckman, Optima XL‐100K; 25 000 × g, 2 h, 4°C), yielding titers of 1–5 × 10^8^ TU/mL. Library representation and integrity were validated by MiSeq sequencing, confirming ≥ 500× coverage with 95% of designed sgRNAs detected.

### 4.3 Isolation and Activation of Primary Human CD8^+^ T Cells

Primary human CD8^+^ T cells were isolated from fresh whole blood obtained from healthy donors within 2 h of collection using the EasySep Human CD8^+^ T Cell Isolation Kit (STEMCELL Technologies, 17953) according to the manufacturer's instructions. This negative‐selection approach ensures high purity of untouched CD8^+^ T cells with minimal ex vivo activation. For activation, CD8^+^ T cells were seeded at 1 × 10^6^ cells/mL in 6‐well (Corning, 3516) or 24‐well (Corning, 3524) plates pre‐coated overnight at 4°C with anti‐CD3 antibody (1 µg/mL; Thermo Fisher Scientific, 16‐0037‐81, RRID: AB_468854). Cells were cultured in complete RPMI‐1640 medium (Gibco, 11875093) supplemented with 10% FBS, soluble anti‐CD3 (1 µg/mL), anti‐CD28 (1 µg/mL; Thermo Fisher Scientific, 16‐0289‐81, RRID: AB_468926), and recombinant human IL‐2 (10 ng/mL; BioLegend, 589102). After 3–5 days of activation, T cells were harvested for subsequent co‐culture experiments.

### 4.4 T Cell Killing Assay

Tumor cells were harvested, resuspended in culture medium at 3 × 10^4^ cells/mL, and seeded at 100 µL per well into 96‐well plates (Corning, CLS3300). Following 24 h of adhesion, CD8^+^ T cells were added at target‐to‐effector (T:E) ratios of 1:1, 1:2, 1:5, and 1:10, and co‐cultures were incubated for 24, 48, and 72 h. At each time point, supernatants were carefully aspirated and wells were washed with 100 µL phosphate‐buffered saline (PBS; Gibco, 70013032) to remove non‐adherent cells and debris. Subsequently, 100 µL of CCK‐8 solution (Vazyme, A311‐02‐AA) was added to each well, and plates were incubated at 37°C for 30 min [68]. Absorbance at 450 nm (OD_450_) was measured using a SpectraMax iD3 microplate reader (Molecular Devices). Tumor cell viability following CD8^+^ T cell‐mediated cytotoxicity was calculated according to the CCK‐8 manufacturer's protocol, which correlates OD_450_ values with the number of viable cells remaining after treatment. This assay enabled quantitative assessment of CD8^+^ T cell cytotoxic activity across multiple T:E ratios and time points.

### 4.5 Degranulation Assay

CD8^+^ T cell degranulation was assessed as previously described [46, 47]. Briefly, CD8^+^ T cells (5 × 10^6^) were co‐cultured with A375‐Cas9 control or miR‐18a KO A375 cells at a T:E ratio of 1:5. At the initiation of co‐culture, FITC‐conjugated anti‐CD107a antibodies (BioLegend, 121606, RRID: AB_572007) were added alone or in combination with 1 µM magrolimab (MCE, HY‐P99029, RRID: AB_3694344). FITC‐conjugated isotype IgG (BioLegend, 400505, RRID: AB_2736919) served as a negative control. After 1 h at 37°C, monensin (1 µm; MCE, HY‐N4302) was added to prevent CD107a internalization, and cells were cultured for a further 2 h. CD8^+^ T cells were then harvested and analyzed by flow cytometry (BD Accuri C6 Plus). The percentage of CD8^+^ CD107a^+^ double‐positive cells was quantified using FlowJo (FlowJo LLC) from three independent biological replicates.

### 4.6 CRISPR KO Screen and Analysis

Stable, single‐cell‐derived A375‐Cas9 and SK‐MEL‐28‐Cas9 cell lines were generated using the LentiCas9‐Blast plasmid (Addgene, #52962). Lentiviral particles were packaged in HEK293FT cells with psPAX2 and pMD2.G packaging plasmids, and virus titers were determined using the Lenti‐Pac HIV qRT‐PCR Titration Kit (Genecopoeia, LT005). A375 and SK‐MEL‐28 cells were transduced at a multiplicity of infection (MOI) of 0.1 for 24 h, followed by selection with 9 µg/mL blasticidin (Thermo Fisher Scientific, 461120) for five days. Single‐cell clones were isolated by limiting dilution [69]. For the CRISPR screen with VEM (Selleckchem, S1267) treatment, we followed established protocols [70, 71, 72]. Briefly, A375‐Cas9 cells were transduced with the pooled sgRNA library at a low MOI (0.3, 500× coverage). At 24 h post‐transduction, puromycin (1 µg/mL; InvivoGen, ant‐pr‐1) was added to select transduced cells. After seven days of selection, cells were divided into three groups: (1) Day 0 control (immediately harvested), (2) Day 23 VEM‐treated, and (3) Day 23 DMSO‐treated (Sigma‐Aldrich, 472301). Genomic DNA was extracted from each group, PCR‐amplified, and subjected to next‐generation sequencing. The screen was performed in independent biological replicates. For the CRISPR screen with CD8^+^ T cell co‐culture, we adapted protocols from previous studies [73, 74, 75, 76]. Briefly, A375‐Cas9 cells were transduced with the pooled sgRNA library (MOI = 0.3, 500× coverage) and selected with puromycin (1 µg/mL) as described above. After seven days, cells were divided into three groups: (1) Day 0 control (harvested and cryopreserved), (2) Day 3 co‐culture (co‐cultured with CD8^+^ T cells for three days), and (3) Day 3 control (cultured alone for three days). CD8^+^ T cells were isolated from three independent healthy donors and pooled to capture a more representative T cell response. Genomic DNA was extracted, PCR‐amplified, and sequenced. This screen was likewise performed in independent biological replicates. Both the VEM resistance and CD8^+^ T cell co‐culture screens were analyzed using the MAGeCK‐VISPR pipeline (v0.5.4) [22]. Positive selection candidates were identified with MAGeCK‐MLE using the criteria *β* > 0 and *P* < 0.05.

### 4.7 Generation of miR‐18a and hnRNP A1 KO Monoclonal Cell Lines

sgRNAs targeting miR‐18a or *HNRNPA1* were cloned into the lentiGuide‐Puro vector (Addgene, #52963) and used to transduce A375‐Cas9, SK‐MEL‐28‐Cas9, Cas9‐expressing A375‐VR, or Cas9‐expressing A375‐TR cells to establish stable knockout lines. Monoclonal populations were subsequently isolated by limiting dilution [69]. Briefly, cells were dissociated with 0.25% EDTA‐free trypsin (Thermo Fisher Scientific, 15050065; 3 min), diluted to 1000 cells/mL in complete DMEM, and seeded into five 96‐well plates by mixing 240 µL of the cell suspension with 100 mL of medium, dispensing 200 µL per well to achieve an average density of approximately 0.5 cells per well. On day 5, wells containing single cell‐derived colonies were identified, marked, and supplemented with 100 µL of fresh medium. After 14 days of expansion, clones reaching >80% confluency were transferred to 24‐well plates. Genomic DNA was extracted from each clone, and the targeted loci were amplified by PCR. Sequencing data were analyzed using CRISPR‐ID [69] with thresholds of ≥50× coverage and <5% background noise. Clones confirmed to carry frameshift mutations in both alleles were selected for subsequent functional validation.

### 4.8 miRNA Mimic Transfection

Melanoma cells were seeded at 2 × 10^5^ cells/mL (2 mL per well) in 6‐well plates. After 24 h, cells were transfected with 100 nM of either miR‐18a‐5p or miR‐18a‐3p mimic. Following an additional 24 h incubation, 3000 cells in 100 µL suspension were seeded into 96‐well plates per experimental group. After a further 24 h, culture medium was supplemented with either VEM or activated CD8^+^ T cells (pre‐stimulated as described above), and cells were incubated for an additional 24 h before viability assessment. Cell viability was measured using the CCK‐8 Cell Counting Kit (Vazyme, A311‐01) according to the manufacturer's instructions. Briefly, culture medium was aspirated and replaced with 100 µL of 10% CCK‐8 solution in fresh medium. Plates were incubated at 37°C for 1 h, and OD_450_ was measured using a microplate reader. Cell viability was expressed as a percentage relative to the control group after background subtraction.

### 4.9 Cell Proliferation Assay

Melanoma cells were seeded at 3000 cells per well in 100 µL medium in 96‐well plates. After 24 h, cells were treated with VEM‐containing medium or co‐cultured with activated CD8^+^ T cells at varying T:E ratios. Cell viability was assessed at 24, 48, and 72 h using the CCK‐8 Cell Counting Kit. Briefly, culture medium was aspirated and replaced with 100 µL of 10% CCK‐8 solution in fresh medium. Plates were incubated at 37°C for 1 h, OD_450_ was measured using a microplate reader (Thermo Fisher Scientific), and cell viability was calculated as a percentage relative to the control group following background subtraction.

### 4.10 Colony Formation Assay

Melanoma cells were seeded in 6‐well plates containing 2 mL of medium per well (500 cells/well for A375 lines; 1,000 cells/well for SK‐MEL‐28 lines), and treated with 1 µm VEM or vehicle control (DMSO). Cells were maintained at 37°C in a humidified atmosphere containing 5% CO_2_, and VEM‐containing medium was replaced every three days with pre‐warmed (37°C) fresh medium to minimize thermal stress. After 12 days, cells were washed with PBS, fixed with 4% paraformaldehyde (Thermo Fisher Scientific, R37814) for 15 min at room temperature (RT), and stained with 0.5% crystal violet (Thermo Fisher Scientific, R40052) for 30 min. Colonies were photographed and counted following extensive washing with distilled water.

### 4.11 Flow Cytometry and Apoptosis Assay

Melanoma cells were seeded at 2 × 10^5^ cells/mL in 6‐well plates (2 mL/well). After 24 h, cells were treated with 1 µm VEM (corresponding to the predetermined IC_50_) or left untreated (DMSO) and incubated at 37°C with 5% CO_2_ for five days, with medium refreshed every 48 h. Adherent cells were washed with ice‐cold PBS, dissociated into single‐cell suspensions using EDTA‐free trypsin (Thermo Fisher Scientific, 15050065), and stained using the Annexin V‐FITC/propidium iodide (PI) Apoptosis Detection Kit (Vazyme, A211‐02). Briefly, harvested cells were washed twice with pre‐chilled PBS and incubated with 5 µL FITC‐labeled Annexin‐V and 5 µL PI Staining Solution for 10 min at RT in the dark. Early apoptotic (Annexin V^+^/PI^−^) and late apoptotic (Annexin V^+^/PI^+^) cell populations were quantified by flow cytometry (BD Accuri C6 Plus) with appropriate compensation controls. Data were analyzed and visualized using FlowJo software (FlowJo LLC).

### 4.12 Generation of VR and TR Cell Lines

VR and TR A375 cell lines were established by stepwise selection. For VR cells, cultures at 70%–80% confluence were initially exposed to 50 nm VEM; drug‐containing medium was replenished every 48 h, and upon the cells reaching 70%–80% confluence, the concentration was sequentially escalated (50 nm → 200 nm → 500 nm → 1 µm → 2 µm). For TR cells, CD8^+^ T cells were added beginning at a T:E ratio of 1:1, with the ratio progressively increased (1:1 → 1:2 → 1:3 → 1:5 → 1:10) following the same confluence‐based medium replacement schedule. Both resistant lines were maintained under their respective final selection pressures for at least two weeks prior to biological validation.

### 4.13 Mature miRNA Quantitative Real‐Time RT‐PCR (RT‐qPCR)

Cells were harvested by centrifugation (1000 × g, 5 min, 4°C), washed with PBS, and lysed in TRIzol reagent (Thermo Fisher Scientific, 15596026). Phase separation was performed by adding 200 µL chloroform (Sigma‐Aldrich, 650498) per 1 mL TRIzol followed by centrifugation (12 000 × g, 12 min, 4°C). RNA was precipitated with isopropanol (Sigma‐Aldrich, 563935), and pellets were washed with 75% ethanol (Sigma‐Aldrich, 493511; 7500 × g, 5 min, 4°C), air‐dried, and resuspended in 20–50 µL diethyl pyrocarbonate (DEPC)‐treated water (MCE, HY‐156262). RNA concentration and purity (A_260_/A_280_ ratio: 1.8–2.1) were assessed using a NanoDrop 2000 spectrophotometer (Thermo Fisher Scientific), and aliquots were stored at −80°C. Mature miRNA expression was quantified using the miRNA 1^st^ Strand cDNA Synthesis Kit (Vazyme, MR‐101) and miRNA Universal SYBR qPCR Master Mix Kit (Vazyme, MQ‐101) according to the manufacturer's instructions. Relative miRNA expression was normalized to *U6* small nuclear RNA (ΔCt = Ct_miRNA_ − Ct_U6_), with fold changes calculated using the 2^−ΔΔCt^ method [77]. Primer sequences are listed in Table S24.

### 4.14 Western Blot

Western blot analysis was performed following standard protocols. Briefly, 2 × 10^6^ cells per sample were lysed in 200 µL of RIPA Lysis and Extraction Buffer (Thermo Fisher Scientific, 89901) supplemented with 1× Protease Inhibitor Cocktail (MCE, HY‐K0010) and phenylmethanesulfonyl fluoride (PMSF; MCE, HY‐B0496). Lysates were incubated on ice for 40 min with intermittent vortexing every 8 min, then centrifuged at 14 000 × g for 20 min at 4°C to pellet cellular debris. Total protein concentrations were determined using a Pierce BCA Protein Assay Kit (Thermo Fisher Scientific, 23235) on a microplate reader (SpectraMax Absorbance Reader, CMax Plus). Samples were normalized to equivalent protein concentrations, mixed with Laemmli Buffer (BIO‐RAD, 1610747), separated by 12% SDS‐PAGE, and transferred onto polyvinylidene difluoride (PVDF) membranes (Millipore, IPFL00010). Membranes were blocked with 5% non‐fat milk (BD‐Difco, 232100) or BSA (Sigma‐Aldrich, A9418) for 1 h at RT (or overnight at 4°C), then incubated with primary antibodies overnight at 4°C. After three washes with TBST buffer (Cell Signaling Technology, 9997S), membranes were incubated with HRP‐conjugated secondary antibodies for 1 h at RT. Following three additional TBST washes, protein bands were visualized using ECL Substrate (Cell Signaling Technology, 6883S) and imaged with a Tanon 5200Multi Imaging System. Primary antibodies used were as follows: phospho‐MEK (Cell Signaling Technology, 3958S, RRID: AB_2138014; 1:1,000), total MEK (Cell Signaling Technology, 4694S, RRID: AB_10695868; 1:2,000), phospho‐AKT (Cell Signaling Technology, 4060S, RRID: AB_2315049; 1:1,000), total AKT (Cell Signaling Technology, 2920S, RRID: AB_1147620; 1:2,000), phospho‐ERK (Cell Signaling Technology, 4377S, RRID: AB_331775; 1:1,000), total ERK (Cell Signaling Technology, 4695S, RRID: AB_390779; 1:2,000), AJUBA (ABclonal, A22039, RRID: AB_3719524; 1:2,000), CD47 (ABclonal, A11382, RRID: AB_2861554; 1:2,000), THBS1 (ABclonal, A2125, RRID: AB_2764144; 1:2,000), and hnRNP A1 (ABclonal, A11564, RRID: AB_2861599; 1:2,000). β‐actin (ABclonal, AC026, RRID: AB_2768234; 1:10,000) or GAPDH (ABclonal, AC001, RRID: AB_2619673; 1:10,000) served as loading controls.

### 4.15 RT‐qPCR

Total RNA was extracted using TRIzol reagent (Thermo Fisher Scientific, 15596026) according to the manufacturer's protocol. RNA samples were treated with DNase I (Thermo Fisher Scientific, EN0521) to eliminate genomic DNA contamination, and RNA integrity was assessed by measuring A_260_/A_280_ ratios using a NanoDrop 2000 spectrophotometer. Approximately 200–500 ng of DNase‐treated RNA per sample was reverse‐transcribed into cDNA using the SuperScript III First‐Strand Synthesis System (Thermo Fisher Scientific, 18080051) with random hexamer primers. Quantitative PCR was performed using SYBR Premix Ex Taq II (Takara, RR820A) on a real‐time PCR system (Thermo Fisher Scientific, Q5 Real‐Time PCR System). Each 20 µL reaction contained 10 µL 2× SYBR Premix Ex Taq II, 0.4 µL of each primer (10 µm), 2 µL cDNA template, and 7.2 µL nuclease‐free water (Sigma‐Aldrich, W4502). Thermal cycling consisted of initial denaturation at 95°C for 30 s, followed by 40 cycles of 95°C for 5 s and 60°C for 30 s. Amplification specificity was verified by melting curve analysis. Relative transcript levels were normalized to *GAPDH* expression and quantified using the 2^−ΔΔCt^ method; error bars represent standard deviations (SDs) from at least three biological replicates. Primer sequences are listed in Table S24.

### 4.16 miR‐18a Target Gene Prediction

RNA‐seq was performed on A375‐Cas9 control and miR‐18a KO cells treated with VEM or co‐cultured with CD8^+^ T cells. Raw sequencing reads were processed using the TOPMed RNA‐seq pipeline [78] to generate gene counts and transcripts per million (TPM) values. DEGs were identified using edgeR [79] with a two‐factor design accounting for genotype (KO versus control) and treatment condition (VEM treatment or CD8^+^ T cell co‐culture). Putative miR‐18a‐5p and miR‐18a‐3p target genes were predicted using TargetScan v7.2 [80] and prioritized by integrating log_2_ fold changes (FC), cumulative weighted context++ scores, and aggregate probability of conserved targeting via the TargetScore package. High‐confidence targets were defined by the following criteria: |log_2_FC| > 0.58 (≥ 1.5‐fold change), *P*‐value < 0.05, and TargetScore > 0.05. This analysis yielded four gene sets representing putative miR‐18a‐5p and miR‐18a‐3p targets associated with VEM resistance and CD8^+^ T cell evasion, respectively.

### 4.17 Pathway Enrichment Analysis

For cell line experiments, DEGs associated with miR‐18a function were identified in the context of VEM resistance and CD8^+^ T cell resistance using the criteria |log_2_FC| ≥ 1 and *P*‐value < 0.05. For patient samples from immunotherapy and targeted cohorts, DEGs were calculated using edgeR's generalized linear model for multi‐timepoint data and the exactTest function for single‐timepoint comparisons. Pathway enrichment analysis was performed using the enrichKEGG function from the clusterProfiler package [81]. The top six significantly enriched pathways, ranked by −log_10_(*P*‐value), were visualized for each comparison. This uniform pipeline enabled cross‐dataset comparison of treatment‐responsive molecular pathways between cell line models and clinical samples.

### 4.18 Sample‐Level miR‐18a Activity Score Estimation

RNA‐seq data were obtained from paired pre‐ and post‐treatment samples across five targeted therapy cohorts (BRAFi/MEKi, *n* = 61 paired samples) [6, 25, 26, 27, 28] and two immunotherapy cohorts (anti‐PD‐1/CTLA‐4, *n* = 59 paired samples) [29, 30], excluding unpaired cases. Raw data were processed using the TOPMed RNA‐seq pipeline to generate quality‐controlled TPM expression matrices. Sample‐level miR‐18a activity was estimated by computing enrichment scores via single‐sample GSEA (ssGSEA) [82] using miR‐18a‐5p/3p target gene sets. For targeted therapy cohorts, VEM resistance‐associated target genes were used; for immunotherapy cohorts, CD8^+^ T cell tolerance‐associated target genes were used. As miRNAs typically suppress target expression, genes upregulated upon miR‐18a loss (log_2_FC ≥ 0.58, *P*‐value < 0.05, TargetScore > 0.05 in miR‐18a KO versus control cells) were selected for enrichment scoring, such that higher target expression reflects reduced miR‐18a activity. To ensure comparability across datasets, cohort‐specific scaling and sign inversion were applied to raw ssGSEA scores calculated for pre‐treatment samples, post‐treatment samples, and their differences (Δ‐score = post‐treatment minus pre‐treatment), such that higher normalized scores correspond to higher inferred miR‐18a activity (i.e., lower target gene expression). These normalized miR‐18a activity scores were used in all downstream analyses.

### 4.19 Sample‐Level Immune Infiltration Analysis

CIBERSORTx [83] was used to estimate immune cell proportions from TPM expression matrices using the LM22 signature matrix (22 immune cell types). Δ‐cell proportions (post‐treatment minus pre‐treatment) were calculated for each patient, and correlations between Δ‐cell proportions of the 22 immune cell types and Δ‐miR‐18a‐5p/3p scores were assessed. Patients were subsequently stratified by RECIST response criteria and by the direction of change in Δ‐miR‐18a‐5p/3p scores (increased versus decreased). Group comparisons were conducted to identify significant differences in Δ‐cell proportions across subgroups.

### 4.20 scRNA‐seq of miR‐18a KO Monoclonal, A375‐VR, and A375‐TR Melanoma Cells

A375 cells at 70%–80% confluence were dissociated into single‐cell suspensions (viability > 90%) and processed for 10× Genomics single‐cell sequencing. Raw sequencing data were aligned and quantified using CellRanger. Quality control was performed in Seurat, filtering out cells with <200 detected genes, <1000 UMI counts, or >20% mitochondrial reads. Doublets and ambient RNA contamination were removed using DoubletFinder [84] and DecontX [61, 85], respectively. Dimensionality reduction was performed by principal component analysis (PCA), and clusters were visualized by uniform manifold approximation and projection (UMAP). Cluster‐specific marker genes (log_2_FC > 0.58, equivalent to ≥1.5‐fold change; adjusted *P* < 0.05) were identified using Seurat's FindAllMarkers function. KEGG pathway enrichment analysis of top marker genes was visualized using the R package ClusterGVis.

### 4.21 Dual Luciferase Reporter Assay

To validate *AJUBA* and *THBS1* as direct miR‐18a targets, wild‐type (WT) and mutant 3′UTR sequences of *AJUBA* and *THBS1* containing predicted miR‐18a binding sites were synthesized by Beijing Genomics Institute (BGI; China), cloned into the pmirGLO dual‐luciferase vector (Promega, E1330), and verified by Sanger sequencing. Recombinant plasmids were quantified using the Qubit dsDNA HS Assay Kit (Thermo Fisher Scientific, Q32851). HEK293FT cells in 24‐well plates at 70%–80% confluence were co‐transfected with equimolar amounts of reporter plasmids (equivalent to 1 µg pmirGLO) and either miR‐18a mimic or negative control (50 nm) using Lipofectamine 2000 (Thermo Fisher Scientific, 11668019). After 24 h at 37°C, luciferase activities were measured using the Dual‐Luciferase Reporter Assay System (Promega, E1960). Relative luminescent signals were determined by normalizing firefly luciferase activity to Renilla luciferase activity.

### 4.22 siRNA‐Mediated Gene Knockdown Assay

Three siRNAs targeting *AJUBA* (#1504, #1383, #1831) or *THBS1* (#438, #1855, #2095), along with scrambled negative control siRNAs, were obtained from GenePharma (Shanghai, China). A375 miR‐18a‐KO cells (3 × 10^5^ cells per well in 6‐well plates) at 70%–80% confluence were transfected using Lipofectamine RNAiMAX (Invitrogen, 13778100). Transfection complexes were prepared by combining siRNA (50 nm final concentration) with transfection reagent at a 1:2.5 ratio in Opti‐MEM (Gibco, 31985062) and incubating for 15–20 min at RT. Complexes were then added to cells and incubated for 6 h, after which the medium was replaced with complete DMEM. Cells were cultured for a further 48 h before analysis. Knockdown efficiency was verified by RT‐qPCR and/or Western blot prior to functional assays. siRNA sequences are listed in Table S24.

### 4.23 Verteporfin and Combination Drug Sensitivity Assays

To evaluate YAP/TAZ pathway inhibition in combination with targeted therapy, melanoma cells were seeded at 3000 cells per well in 96‐well plates. After 24 h, cells were treated with VEM (1 or 2 µm) alone or in combination with verteporfin (MCE, HY‐B0146; 0.2 µm), VT3989 (Selleck, E6486; 0.2 µm), trametinib (MCE, HY‐10999; 10 nm), or ulixertinib (MCE, HY‐15816; 2 µm) for 24 h at 37°C. Cell viability was assessed by CCK‐8 assay: culture medium was replaced with DMEM containing 10% CCK‐8 reagent (100 µL per well) and incubated for 1 h at 37°C, after which OD_450_ was measured using a SpectraMax iD3 microplate reader (Molecular Devices). Cell viability was calculated relative to vehicle‐treated Cas9‐control cells (set as 100%).

### 4.24 Cell‐Derived Xenograft (CDX) Model

A375 miR‐18a KO cells were cultured in DMEM supplemented with 10% FBS at 37°C in 5% CO_2_. Upon reaching 90% confluence, cells were harvested by trypsinization, centrifuged, and resuspended to 2 × 10^7^ cells/mL (viability > 95%); the suspension was maintained on ice until injection. Sixty female BALB/c‐Nude mice (4–5 weeks old, SPF‐housed) were randomly assigned to six experimental groups (*n* = 10 mice per group): (1) control, (2) VEM alone, (3) miR‐18a‐5p mimics alone, (4) VEM + miR‐18a‐5p mimics, (5) VP alone, and (6) VEM + VP. VEM was dissolved in DMSO, and VP was dissolved in CMC‐Na according to the respective manufacturer's instructions; the control group received equivalent volumes of DMSO and/or CMC‐Na. Each mouse received a subcutaneous injection of 100 µL cell suspension into the iodine‐disinfected left inguinal region using a 1 mL syringe (BD, 300841), with the injection site stabilized by toothless forceps during needle withdrawal to minimize cell leakage. Tumor volume was calculated as length × width^2^/2 and measured twice weekly. When tumors reached approximately 100 mm^3^, treatments were administered every three days for 14 days: miR‐18a‐5p mimics (intratumoral injection), VEM (oral gavage), and VP (intraperitoneal injection). Mice were euthanized upon reaching humane endpoints (tumor volume ≥ 1500 mm^3^ or ulceration). Tumors were excised, photographed, and fixed in paraformaldehyde for immunohistochemical analysis.

### 4.25 Immunohistochemistry (IHC)

FFPE tumor sections from all CDX treatment groups (control, VEM, miR‐18a‐5p mimics, VEM + miR‐18a‐5p mimics, VP, and VEM + VP) were immunostained for AJUBA, Ki67, and YAP. FFPE tissues from C57BL/6 mouse cohorts (*n* = 7 per group; control versus miR‐18a‐5p mimic‐treated) were analyzed for Ki67, Thbs1, and Cd47. Tissue microarrays (TMAs) from 53 melanoma patient samples were evaluated for hnRNP A1 expression. Slides were baked at 60°C for 1 h, deparaffinized in xylene (Sigma‐Aldrich, 534056; two changes, 30 min each), and rehydrated through a graded ethanol series (100% twice for 10 min each; 95%, 80%, and 70% for 5 min each), followed by rinsing in distilled water. Antigen retrieval was performed in 2 L of citrate buffer (pH 6.0) using a pressure cooker (95°C, 10 min). Endogenous peroxidase activity was quenched with 3% H_2_O_2_ (Sigma‐Aldrich, 323381) in methanol (30 min, RT), and nonspecific binding was blocked with 5% BSA (1 h, 37°C). After PBS washes, sections were incubated overnight at 4°C with the following primary antibodies: AJUBA (Abcam, ab244285, RRID: AB_3719522; 1:100), Ki67 (Abcam, ab16667, RRID: AB_302459; 1:200), YAP (Cell Signaling Technology, 14074, RRID: AB_2650491; 1:400), THBS1 (Abcam, ab267388, RRID: AB_3271579; 1:100), CD47 (Affinity, DF6649, RRID: AB_2838611; 1:100), and hnRNP A1 (Affinity, AF5268, RRID: AB_2837754; 1:100). After washing, slides were incubated with HRP‐conjugated goat anti‐rabbit (Abclonal, AS014, RRID: AB_2769854; 1:100) or goat anti‐mouse (Abclonal, AS003, RRID: AB_2769851; 1:100) secondary antibodies for 30 min at RT. Immunoreactivity was visualized using 3,3′‐diaminobenzidine (DAB) substrate under microscopic monitoring (1–5 min), followed by hematoxylin counterstaining. Slides were dehydrated through an ascending ethanol‐xylene series and mounted with neutral balsam. Staining intensity was independently scored by two blinded pathologists using a semi‐quantitative scoring system based on staining intensity and the percentage of positive tumor cells. For quantitative image analysis, digital images were acquired at 200× magnification under standardized conditions and analyzed using ImageJ software (v1.53t, NIH). DAB signals were isolated by color deconvolution (H‐DAB mode). Regions of interest (ROIs) encompassing tumor areas were defined, and a uniform optical density threshold (≥0.2) was applied to exclude background. The percentage of DAB‐positive area (area fraction, %) within each ROI was quantified by pixel‐based densitometry.

### 4.26 Melanoma Syngeneic Graft Mouse Model

miR‐18a KO YUMM1.7 cells were cultured in DMEM/F‐12 supplemented with 10% FBS to 90% confluence, harvested by trypsinization, and resuspended to 5 × 10^7^ cells/mL; the suspension was maintained on ice until use. Twenty female C57BL/6 mice (4–5 weeks old) were randomly divided into two groups (*n* = 10 per group): control and miR‐18a‐5p mimics. Under sterile conditions, 100 µL of cell suspension (5 × 10^6^ cells) was subcutaneously injected into the iodine‐disinfected left inguinal region using a 1 mL syringe, with the injection site stabilized by toothless forceps during needle withdrawal to prevent leakage. Four days post‐injection, 7 mice per group with matched baseline tumor volumes (50 ± 5 mm^3^) were selected for treatment. Mice received intratumoral injections every two days as follows: control group (20 µL DEPC‐treated water) or miR‐18a‐5p mimics group (20 µL DEPC‐treated water containing 2 OD units of miR‐18a‐5p mimics) [86]. For the Cd47 blockade experiment, miR‐18a KO YUMM1.7 cells were prepared at 5 × 10^7^ cells/mL, and 30 four‐to‐five‐week‐old C57BL/6 mice were inoculated subcutaneously with 5 × 10^6^ cells (100 µL) in the left inguinal region. Four days post‐inoculation, mice with consistent baseline tumor volumes were randomly assigned to three treatment groups (*n* = 7 per group): (1) anti‐CD47 antibody (400 µg in 200 µL PBS; BP0283, Bio X Cell, RRID: AB_3720020), (2) isotype control antibody (400 µg in 200 µL PBS; A2106, Selleck, RRID: AB_3722689), or (3) PBS alone (200 µL). All treatments were administered intraperitoneally every two days. Tumor volume was recorded at each treatment timepoint until mice reached humane endpoints (maximum allowable tumor volume). Upon sacrifice, tumors were excised and processed for histological or molecular analysis.

### 4.27 scRNA‐Seq Analysis of Engrafted Tumors

Sequencing reads were aligned to the mm10 reference genome and quantified using SeekSoulTools (–chemistry DD5V1). Quality control was performed in Seurat, excluding cells with <200 detected genes, <1000 UMIs, or >20% mitochondrial reads. Doublets were identified and removed using DoubletFinder, and ambient RNA contamination was corrected with DecontX (celda package). Batch effects were corrected using Seurat's integration workflow before PCA and UMAP clustering. Differential gene expression between miR‐18a‐5p mimic and control groups was assessed using MAST [87]. Hallmark pathway enrichment was evaluated using fgseaMultilevel [88], and intercellular communication dynamics were analyzed with CellChat [89].

### 4.28 Multiplex Fluorescence IHC

Frozen OCT‐embedded tissue sections (10 µm) were subjected to multiplex immunofluorescence staining [90] using a four‐color TSA‐Rab‐275 Kit (Panovue, 100020). The following primary antibodies were used: anti‐THBS1 (Abcam, ab267388, RRID: AB_3271579; 1:100), anti‐CD47 (Affinity, DF6649, RRID: AB_2838611; 1:100), and anti‐CD8a (Proteintech, 29896‐1‐AP, RRID: AB_2935485; 1:100). Sections were air‐dried for 30 min at RT, washed three times in PBST (5 min each), and treated with 3% H_2_O_2_ (two washes, 5 min each) to quench endogenous peroxidase activity. Blocking was performed in a humidified chamber (two cycles, 10 min each). Primary antibodies (50 µL per section) were applied and incubated overnight at 4°C, followed by HRP‐conjugated secondary antibodies (30 min, RT). TSA dyes (1:100) were applied for 10 min in the dark. Antibody stripping (20 min) enabled sequential staining cycles. Nuclei were counterstained with DAPI (1:100 in TBST) for 15 min in the dark. Slides were mounted with anti‐fade medium, coverslipped without air bubbles, sealed with resin, and allowed to dry in the dark for 1 h before imaging. Single‐stained and unstained controls were included for spectral unmixing. Images were acquired using a Zeiss LSM 980 confocal microscope with a 20×/0.8 NA objective. Staining results were independently scored by two blinded observers trained on the same criteria: tumor cell staining intensity was graded as 0 (no staining), 1 (light yellow), 2 (brownish‐yellow), or 3 (dark brown); the percentage of positively stained tumor cells was scored as 0 (0%), 1 (<25%), 2 (25%–49%), 3 (50%–74%), or 4 (≥75%). The final score (sum of intensity and percentage scores; range 0–7) was categorized as low expression (0–3) or high expression (4–7). After independent scoring, the two observers' assessments were found to be entirely concordant across all cases, indicating excellent interobserver agreement.

### 4.29 RNA Immunoprecipitation (RIP) Assay

Cells were lysed in RIP buffer (150 mm KCl [Sigma‐Aldrich, P5405], 25 mm Tris pH 7.5 [Thermo Fisher Scientific, 15567027], 0.5 mm DTT [Sigma‐Aldrich, D9779], 0.5% NP‐40 [Thermo Fisher Scientific, FNN0021], 1 mm PMSF [Roche, 10837091001], 1× protease inhibitors [Thermo Fisher Scientific, 78425]) for 30 min on ice. Lysates were cleared by centrifugation (14 000 × g, 10 min, 4°C). Supernatants were incubated with pre‐equilibrated Protein A/G magnetic beads (MCE, HY‐K0202) conjugated to anti‐hnRNP A1 antibody (Abcam, ab208029, RRID: AB_3719521) for 6 h at 4°C with rotation, followed by overnight incubation. Beads were pelleted (1000 × g, 10 s) and washed twice with low‐salt buffer (50 mm Tris pH 7.5, 150 mm NaCl [Sigma‐Aldrich, S7653], 1 mm MgCl_2_ [Sigma‐Aldrich, M2670], 0.5% NP‐40, 2 mm EDTA [Thermo Fisher Scientific, 15575020], 1 mM DTT, 100 U/mL RNasin Ribonuclease Inhibitor [Promega, N2615], 1× protease inhibitors) and twice with high‐salt buffer (identical composition except 300 mm NaCl); each wash was performed for 5 min at 4°C. Bead‐bound complexes were digested with Proteinase K (Beyotime, ST535) at 55°C for 10 min. RNA was extracted using TRIzol reagent with glycogen (Invitrogen, AM9516) as a carrier, treated with DNase I, and analyzed by RT‐qPCR. Isotype‐matched IgG (Cell Signaling Technology, 2729S, RRID: AB_1031062) and nuclease‐free water served as negative controls to confirm specificity.

### 4.30 Stable Overexpression of hnRNP A1 and AJUBA

Stable hnRNP A1‐ or AJUBA‐overexpressing melanoma cell lines were established using a lentiviral delivery system. Briefly, the full‐length coding sequences of human hnRNP A1 and AJUBA were synthesized by BGI and individually cloned into the pLVX‐FLAG‐puro lentiviral vector (Qincheng BIO, China). Lentiviral particles were produced in HEK293T packaging cells following the standard protocols described above. Culture supernatants were harvested 48 h post‐transfection and clarified by filtration through 0.45 µm membranes (Millipore, HPWP01300). A375, SK‐MEL‐28, A375‐VR, or A375‐TR cells were transduced with the lentivirus at an MOI of 0.1 in the presence of 8 µg/mL polybrene (Beyotime, C0351). At 96 h post‐transduction, puromycin selection (5 µg/mL, a concentration optimized by 5‐day dose‐response assays) was initiated. After four days of selection, surviving cell populations were expanded from 96‐well plates to 6‐well plates. Stable overexpression of hnRNP A1 or AJUBA was confirmed by RT‐qPCR and Western blot analyses.

### 4.31 CISH for miR‐18a‐5p

FFPE tissues from 53 melanoma patients who received targeted therapy or immunotherapy at Tianjin Medical University Cancer Hospital (April 2019–February 2023) were collected. Tumor regions were delineated on hematoxylin and eosin (H&E)‐stained slides to guide TMA construction. TMA sections were deparaffinized by baking at 60°C for 1 h, followed by two 10 min xylene washes and rehydration through graded ethanol (100%, 95%, 80%, 70%; 5 min each). Endogenous peroxidase activity was quenched with 3% H_2_O_2_ for 10 min at RT. Nucleic acids were exposed by pepsin‐citric acid digestion (37°C, 15 min), followed by fixation in 4% formaldehyde (10 min). After pre‐hybridization at 37°C for 1 h, sections were incubated overnight at 37°C with 200 µL digoxigenin‐labeled hsa‐miR‐18a‐5p probes (Boster, MK1030). Post‐hybridization stringency washes were performed using graded SSC buffers (Yeasen, 60142es76; 2× to 0.2×) at 37°C, followed by blocking and sequential incubation with biotinylated anti‐digoxigenin antibody (Abcam, ab419, RRID: AB_304356; 1:200, 1 h) and streptavidin‐biotin complex (Boster, SA1078; 1:100, 20 min), with PBS washes between steps. Signal was detected by DAB chromogen under microscopic monitoring, followed by hematoxylin counterstaining (Sigma‐Aldrich, 2852), dehydration through graded ethanol and xylene, and mounting with Permount Mounting Medium (Thermo Fisher Scientific, SP15‐100). Expression was independently scored by two blinded observers using a composite scoring system: staining intensity was graded as 0 (negative), 1 (weak, faint yellow), 2 (moderate, brown‐yellow), or 3 (strong, brown‐black); the percentage of positive tumor cells was scored as 0 (0%), 1 (<25%), 2 (25%–49%), 3 (50%–74%), or 4 (≥75%). Final scores (sum of intensity and percentage scores; range 0–7) were categorized as low expression (0–3) or high expression (4–7).

### 4.32 Ethics Declarations

This study involving human participants, including the use of melanoma patient tissue specimens and clinical data, was approved by the Ethics Committee of Tianjin Medical University Cancer Institute and Hospital (approval number: bc2022110). Written informed consent was obtained from all participants in accordance with institutional guidelines and the Declaration of Helsinki. All animal experiments were conducted in compliance with institutional and national guidelines for the care and use of laboratory animals and were approved by the Animal Ethics Committee of Tianjin Medical University Cancer Institute and Hospital (approval number: WSJK‐AE‐2022004).

### 4.33 Statistical Analysis

All in silico statistical analyses were performed using R version 4.3.1 (www.r‐project.org). For datasets with *n* ≥ 6, normality was assessed using the Shapiro‐Wilk test. No outliers were excluded from the analyses. All data are presented as mean ± standard deviation (SD) unless otherwise stated, and the exact sample size (n) for each analysis is provided in the corresponding figure legends. Statistical analyses were performed using GraphPad Prism 9.0 (GraphPad Software, San Diego, CA, USA). For comparisons between two independent groups, an unpaired two‐tailed Student's *t*‐test was used for normally distributed data, while the Mann‐Whitney U test was applied for non‐normally distributed data or when n < 6. Correlations between variables were assessed using Pearson or Spearman correlation analysis with the cor.test function in R. For multiple group comparisons, two‐way analysis of variance (ANOVA) was performed, followed by Tukey's post hoc test for pairwise comparisons. A two‐tailed significance level (alpha) of 0.05 was used for all statistical tests, and *P* values < 0.05 were considered statistically significant. All mice were included in the analyses unless technical failures occurred (e.g., equipment malfunction).

## Author Contributions

Z.W., H.T.L., X.Q.W., and J.J.T. contributed equally to this work. M.J.L., Z.W., J.L.Y., and D.D.H. designed the studies and wrote the manuscript. D.D.H., H.T.L., J.J.T., and Z.W. designed and prepared all figures and supplementary figures. Z.W., H.T.L., X.Q.W., J.J.T., J.Y.Z., W.Y.X., Q.L., J.X.L., X.M.J., X.F., H.Z.C., T.L.Y., L.X.W., and K.Z. performed the experiments. D.D.H., M.H.L., and W.L.Y. conducted the bioinformatics and data analysis. M.J.L., Z.W., and J.L.Y. contributed scientific expertise, reviewed the manuscript, and supervised the study. All authors approved the final version of the manuscript.

## Conflicts of Interest

The authors declare no conflicts of interest.

## Supporting information




**Supporting File 1**: advs75832‐sup‐0001‐SuppMat.docx.

**Supporting File 2**: advs75832‐sup‐0002‐Table S1‐S24.xlsx.

## Data Availability

The raw sequence data reported in this paper have been deposited in SRA under BioProject PRJNA1223135. All sequencing data generated in this study have now been deposited in the National Center for Biotechnology Information (NCBI) Gene Expression Omnibus (GEO) database to ensure full public accessibility. Specifically, the bulk RNA‐seq data are available under accession number GSE318354, the scRNA‐seq data under GSE318353, and the CRISPR screen data under GSE318501. The other data that support the findings of this study are available from the corresponding author upon reasonable request.
