# Supplementary material for: Genome‐Wide CRISPR Screen Identifies a microRNA Orchestrating Pleiotropic Resistance to Targeted Therapy and T Cell Immunity in Melanoma
Source: Adv Sci (Weinh). 2026 May 26;13(47):e15158. doi: 10.1002/advs.202515158 (PMC13335869; doi:10.1002/advs.202515158)
Supplement: Supplementary file 1 [file ADVS-13-e15158-s002.docx]

**SUPPLEMENTAL FIGURE LEGENDS**

**FIGURE** **S1** **|** miRNA-targeted CRISPR library design and miR-18a KO strategy. A) Composition of miRNA-targeted CRISPR libraries. B) Correlation matrix showing correlations of sgRNA read counts between biological replicates in VEM treatment and CD8^+^ T cell co-culture screenings. C) Schematic of the miR-18a WT locus in A375-Cas9 (Control) cells and sequence alignment of three A375-Cas9 KO clones (#1-3). D) Expression levels of miR-18a-5p/3p in A375-Cas9 (control), CRISPR/Cas9 KO pooled population (mixed), and single clones (#1-3), normalized to hU6. E) Schematic of the miR-18a WT locus in SK-MEL-28-Cas9 (Control) cells and sequence alignment of three SK-MEL-28-Cas9 KO clones (#1-3). F) Expression levels of miR-18a-5p/3p in SK-MEL-28-Cas9 (control), CRISPR/Cas9 KO mixed, and single clones (#1-3), normalized to hU6. G, H) Cell proliferation at 48 h (G) and 72 h (H) post-VEM treatment in miR-18a KO A375 and SK-MEL-28 cells. Control: A375-Cas9 or SK-MEL-28-Cas9 cells. Data represent means ± SD; n = 3 biologically independent samples for panels D and F, and n = 4 for panels G and H. Unpaired two-tailed Student's *t*-test is used to calculate *P*-values in panels D, F, G, and H: **, *P* < 0.01; ***, *P* < 0.001; ****, *P* < 0.0001; ns, not significant.

**FIGURE** **S2** **|** Impact of miR-18a KO on melanoma cell response to VEM, cell survival, colony formation, and apoptosis. A) VEM dose-response curves in miR-18a KO clone #1 A375 and SK-MEL-28 cells. B) CD8^+^ T cell ratio-dependent cytotoxicity curves in miR-18a KO clone #2 A375 and SK-MEL-28 cells. Control: A375-Cas9 or SK-MEL-28-Cas9 cells. C) Cell proliferation in A375-Cas9 (control) and miR-18a KO cells under MAPK inhibitor (1 μM VEM, 10 nM trametinib [TMT], 10 nM TMT + 1 μM VEM) or ERK inhibitor (2 μM ulixertinib [BVD-523], 2 μM BVD-523 + 1 μM VEM) treatments. D) Cell proliferation in SK-MEL-28-Cas9 (control) and miR-18a KO cells under MAPK/ERK inhibitor treatments. E, F) Colony formation assay in A375-Cas9 (E) and SK-MEL-28-Cas9 (F) control and miR-18a KO clones (#1-3) with or without VEM treatment. G, H) Apoptosis analysis (Annexin V/PI staining) in A375-Cas9 (G) and SK-MEL-28-Cas9 (H) control and miR-18a KO clones (#1-3) with or without VEM treatment. I) Quantification of colony formation numbers in A375-Cas9 and SK-MEL-28-Cas9 control and miR-18a KO clones (#1-3) with or without VEM. J) Quantification of apoptotic rates (%) in A375-Cas9 and SK-MEL-28-Cas9 control and miR-18a KO clones (#1-3) with or without VEM. Data represent means ± SD; n = 4 biologically independent samples for panels A–D, and n = 3 for panels I and J. Unpaired two-tailed Student's *t*-test is used to calculate *P*-values in panels A–D, I, and J: **, *P* < 0.01; ***, *P* < 0.001; ****, *P* < 0.0001; ns, not significant.

**FIGURE** **S3** **|** Negative association between endogenous miR-18a-5p expression and RAF/MEK inhibitor sensitivity. A–C) Scatter plots show the relationship between basal miR-18a-5p levels (log_2_, x-axis) and area under the dose–response curve (AUC, y-axis) for dabrafenib (A), RAF265 (B), and regorafenib (C) across cancer cell lines. Each dot represents one cell line; lines indicate linear regression with 95% confidence intervals, with Spearman correlation coefficients (ρ) and *P*-values as indicated.

**FIGURE** **S4** **|** Genomic and clinical implications of *MIR17HG* locus alterations in melanoma. A) Distribution of point mutation status at the *MIR17HG* locus across melanoma subtypes ("No mutation" versus "Not profiled"). B) Copy number variation status at the *MIR17HG* locus and association with patient progression-free survival (months).

**FIGURE** **S5** **|** Transcriptomic analysis of miR-18a KO versus A375-Cas9 (control) cells. A) PCA and hierarchical clustering of RNA-seq data comparing miR-18a KO versus control A375 cells treated with VEM (left) or co-cultured with CD8^+^ T cells (right). B) Volcano plots showing DEGs in miR-18a KO versus control under VEM (left) and CD8^+^ T cell co-culture (right). C) Schematic illustrating the approach to identify miR-18a 5p/3p target genes under VEM and CD8^+^ T cell treatments.

**FIGURE** **S6** **|** Overview of clinical cohorts and miR-18a stratification. A) Tabulated summary of systematically curated RNA-seq or Beadchip data from paired patient samples for targeted therapy and immunotherapy cohorts. B, C) PCA comparing RNA-seq data from targeted therapy (B) and immunotherapy cohorts (C). D–G) Patients from targeted therapy (D, E) and immunotherapy (F, G) cohorts were ordered by Δ-miR-18a-5p/3p scores (Δ = post-treatment minus pre-treatment) and categorized based on high or low pre-miR-18a-5p/3p scores.

**FIGURE** **S7** **|** miR-18a-3p and miR-18a-5p dynamics in relation to clinical outcomes and RECIST responses. A) Association of Δ-miR-18a-3p scores with RECIST responses (CR = complete response, PR = partial response, SD = stable disease, PD = progressive disease) (left) and progression-free survival (PFS) (right) in targeted therapy cohorts, categorized by the pre-miR-18a-3p score and Δ-miR-18a-3p score changes (Δ = post-treatment minus pre-treatment). B) Association of Δ-miR-18a-3p scores with RECIST responses (left) and overall survival (OS) (right) in immunotherapy cohorts, categorized by the pre-miR-18a-3p score and Δ-miR-18a-3p score changes (Δ = post-treatment minus pre-treatment). C) Forest plot showing hazard ratios for PFS in targeted therapy cohorts based on Δ-miR-18a-5p, stratified by pre-miR-18a-5p score, with stepwise adjustment for clinical covariates. D) Forest plot showing hazard ratios for PFS in targeted therapy cohorts based on Δ-miR-18a-3p, stratified by pre-miR-18a-3p score, with stepwise adjustment for clinical covariates. E) Forest plot showing hazard ratios for OS in immunotherapy cohorts based on Δ-miR-18a-5p, stratified by pre-miR-18a-5p score, with stepwise adjustment for clinical covariates. F) Forest plot showing hazard ratios for OS in immunotherapy cohorts based on Δ-miR-18a-3p, stratified by pre-miR-18a-3p score, with stepwise adjustment for clinical covariates. Abbreviations: Tx, treatment; Sex, sex; M, metastasis status.

**FIGURE** **S8** **|** Interactions between miR-18a dynamics and immune cell infiltration. A) Correlations between Δ-miR-18a-5p/3p scores and Δ-proportions in immune cell types (Δ = post-treatment minus pre-treatment). B, C) Comparison of Δ-proportions of M1 macrophage (B) and M2 macrophage (C) between patients with increased and decreased Δ-miR-18a-5p scores. D–G) Comparison of Δ-proportions of CD8^+^ T cells (D), CD4^+^ memory T cells (E), M1 macrophage (F), and M2 macrophage (G) between patients with increased and decreased Δ-miR-18a-3p scores.

**FIGURE** **S9** **|** Impact of miR-18a KO on MAPK/AKT signaling and resistance pathways. A, B) Western blot of MAPK/ERK and PI3K–AKT pathway activation in A375-Cas9 (control) and miR-18a KO A375 cells following VEM treatment (A) or VEM + trametinib (TMT) treatment (B). C) Western blot of MAPK/ERK and PI3K–AKT pathway activation in A375-Cas9 (control) and miR-18a KO A375 cells co-cultured with CD8^+^ T cells. D, E) Western blot of MAPK/ERK and PI3K–AKT pathway activation in SK-MEL-28-Cas9 (control) and miR-18a KO SK-MEL-28 cells following VEM treatment (D) or VEM + TMT (E). F) Western blot of MAPK/ERK and PI3K–AKT pathway activation in SK-MEL-28-Cas9 (control) and miR-18a KO SK-MEL-28 cells co-cultured with CD8^+^ T cells. G, H) Volcano plots depicting differentially expressed genes (DEGs) between A375-VR and A375 control (G) cells or A375-TR and A375 control (H) cells.

**FIGURE** **S10** **|** Single-cell profiling and quality control of A375 Control, miR-18a-KO, VR, and TR cells. A) Violin plots showing genes detected per cell in Control, miR-18a KO, A375-VR, and A375-TR populations. B) Violin plots showing total UMIs per cell across the four conditions. C) Violin plots showing mitochondrial gene percentage per cell. D) Bar plot showing the number of cells in each cluster (C0–C12). E) Dot plots showing, for each cluster (C0–C12), the number of differentially expressed genes in miR-18a-KO, A375-VR, and A375-TR cells relative to Control.

**FIGURE** **S11** **|** Regulation of *AJUBA* expression and tumor growth by miR-18a KO. A) Schematic of *AJUBA* 3′UTR luciferase reporter constructs with WT and MUT miR-18a binding sites. B) *AJUBA* mRNA levels in miR-18a KO versus A375-Cas9 (control) cells. C) Western blot of AJUBA protein in miR-18a KO A375 cells treated with si*AJUBA* and VEM. D) Bar plot illustrating proliferation rates of A375 cells under conditions of NC (vehicle control), 2 µM VEM, 0.2 µM YAP/TEAD inhibitor VT3989 (VT), and their combination (2 µM VEM + 0.2 µM VT). E) Western blot validation of AJUBA overexpression and cell proliferation assay in A375 cells treated with or without VEM. F) Western blot validation of AJUBA overexpression and cell proliferation assay in SK-MEL-28 cells treated with or without VEM. G) RT-qPCR analysis showing that miR-18a KO upregulates *CTGF* and *CYR61* mRNA expression in A375 cells treated with or without VEM. H) *AJUBA* knockdown rescues *CTGF* and *CYR61* mRNA upregulation induced by miR-18a KO in A375 cells treated with or without VEM. A375-Cas9 cells served as controls, and DMSO (0 μM) was used as the vehicle control. I) Tumor weight under various treatments: vehicle treatment (control), VEM, miR-18a-5p mimic, VEM + miR-18a-5p mimic, verteporfin (VP), and VEM + VP. J) Statistical results of YAP nuclear-cytoplasmic ratio. Data represent means ± SD; n = 3 biologically independent samples for panels B, G, and H; n = 4 for panels D–F; and n = 7 for panels I and J. Unpaired two-tailed Student's *t*-test is used to calculate *P*-values in panels B and D–J: *, *P* < 0.05; **, *P* < 0.01; ***, *P* < 0.001; ****, *P* < 0.0001; ns, not significant.

**FIGURE** **S12** **|** miR-18a regulates the Thbs1-Cd47 axis to modulate tumor immunity. A) Schematic of *THBS1* 3′UTR luciferase reporter with WT and MUT miR-18a binding sites. B) *THBS1* mRNA levels in A375-Cas9 (control) versus miR-18a KO cells. C, D) Schematic of CRISPR/Cas9-mediated miR-18a KO in YUMM1.7 cells (C) and validation of miR-18a expression in YUMM1.7-Cas9 (control) versus KO clones (D). E) Tumor weight in mice treated with miR-18a-5p mimic versus control. F–H) scRNA-seq quality control: UMIs per cell (F), genes per cell (G), and mitochondrial content (H). I) Dot plot of marker gene expression across cell types in tumor tissues. J) Cell type-specific DEGs between miR-18a-5p mimic and control treatments. K) Cell-cell communication networks showing Thbs signaling patterns in control versus miR-18a-5p mimic-treated mice. L) Western blot of CD47 and β-actin in CD8^+^ T cells treated with increasing magrolimab concentrations. M) Multiplex IHC of Thbs1, Cd47, and Cd8a in tumor sections from control and miR-18a-5p mimic-treated C57BL/6 mice. N) Flow cytometry analysis of CD107a degranulation in activated CD8^+^ T cells co-cultured with A375-Cas9 (Control) or miR-18a-KO cells in the presence of magrolimab (CD47i) or isotype IgG (T:E = 1:5), with quantification shown. O–R) Schematic illustration of the Cd47 blockade treatment protocol (O). C57BL/6 mice were subcutaneously inoculated with miR-18a KO YUMM1.7 cells and subsequently treated with PBS alone, anti-CD47 antibody (Cd47i, 400 μg), or isotype IgG control (400 μg) via intraperitoneal injection (100 μL per dose). Tumor images (P), tumor growth curves (Q), and endpoint tumor weights (R) are shown. Data represent means ± SD; n = 3 biologically independent samples for panels B, D and N, and n = 7 for panels E, Q, and R. Two-way ANOVA followed by Tukey's post hoc test is used to calculate *P*-values in panel Q and unpaired two-tailed Student's *t*-test is used to calculate *P*-values in panels B, D, E, N, and R: *, *P* < 0.05; **, *P* < 0.01; ***, *P* < 0.001; ****, *P* < 0.0001; ns, not significant.

**FIGURE** **S13** **|** hnRNP A1 promotes VEM resistance and activated CD8^+^ T cell evasion in A375-VR and A375-TR cells. A, B) Western blot validation of *HNRNPA1*-KO and rescue in A375-VR (A) and A375-TR (B) cells. C, D) RT-qPCR analysis of miR-18a expression in *HNRNPA1*-KO and rescue A375-VR (C) and A375-TR (D) cells. E) Cell proliferation assay assessing VEM resistance in *HNRNPA1*-KO and rescue A375-VR cells. F) Activated CD8^+^ T cell-mediated killing assay in *HNRNPA1*-KO and rescue A375-TR cells. Data are represented as the means ± SD; n = 3 biologically independent samples for panels C and D, and n = 4 for panels E and F. Unpaired two-tailed Student's *t*-test is used to calculate *P*-values in panels C–F: **, *P* < 0.01; ***, *P* < 0.001; ****, *P* < 0.0001; ns, not significant.

**FIGURE** **S14** **|** Vemurafenib treatment and activated CD8^+^ T cell co-culture decrease *HNRNPA1* and miR-18a expression in melanoma cells. A, B) *HNRNPA1* mRNA (A) and protein (B) expression levels in A375 cells treated with or without VEM (1 μM). C) miR-18a expression levels in A375 cells treated with or without VEM (1 μM). D, E) *HNRNPA1* mRNA (D) and protein (E) expression levels in SK-MEL-28 cells treated with or without VEM (1 μM). F) miR-18a expression levels in SK-MEL-28 cells treated with or without VEM (1 μM). G, H) *HNRNPA1* mRNA (G) and protein (H) expression levels in A375 cells co-cultured with or without activated CD8^+^ T cells (T:E ratio = 1:5). I) miR-18a expression levels in A375 cells co-cultured with or without activated CD8^+^ T cells (T:E ratio = 1:5). Data are represented as the means ± SD; n = 3 biologically independent samples for panels A–I. Unpaired two-tailed Student's *t*-test is used to calculate *P*-values in panels A–I: *, *P* < 0.05; **, *P* < 0.01; ***, *P* < 0.001.

**FIGURE** **S15** **|** miR-18a does not affect *HNRNPA1* expression. A) RT-qPCR analysis of *HNRNPA1* mRNA levels in miR-18a KO A375 cells and control (A375-Cas9) cells treated with or without VEM (1 μM) and activated CD8^+^ T cells (T:E ratio = 1:5). B) RT-qPCR analysis of *HNRNPA1* mRNA levels in miR-18a KO SK-MEL-28 cells and control (SK-MEL-28-Cas9) cells treated with or without VEM (1 μM). C) Western blot analysis of hnRNP A1 protein levels in miR-18a-KO A375 cells and control (A375-Cas9) cells. D) Western blot analysis of hnRNP A1 protein levels in miR-18a-KO SK-MEL-28 cells and control (SK-MEL-28-Cas9) cells. Data are represented as the means ± SD; n = 3 biologically independent samples for panels A–D. Unpaired two-tailed Student's *t*-test is used to calculate *P*-values in panels A–D: ns, not significant.

**FIGURE** **S16** **|** Detection of hnRNP A1 and miR-18a in melanoma patient specimens. A) Representative IHC staining of hnRNP A1 in melanoma tissues showing high versus low expression. B) Representative CISH staining of miR-18a in melanoma tissues showing high versus low expression.

**TABLE LEGENDS**

**TABLE** **S1** **|** CRISPR/Cas9 sgRNA library targeting key regions of pre-miRNA sequence.

**TABLE S2** **|** miRNA-level analysis of differential sgRNA abundance of CRISPR KO screen under VEM treatment versus control.

**TABLE S3** **|** sgRNA-level abundance of CRISPR KO screen under VEM treatment versus control across replicates.

**TABLE S4** **|** miRNA-level analysis of differential sgRNA abundance of CRISPR KO screen in CD8^+^ T cell co-culture versus control.

**TABLE S5** **|** sgRNA-level abundance of CRISPR KO screen in CD8^+^ T cell co-culture versus control across replicates.

**TABLE S6** **|** DEGs associated with miR-18a KO under VEM treatment.

**TABLE S7** **|** DEGs associated with miR-18a KO under CD8^+^ T cell co-culture.

**TABLE S8** **|** Predicted miR-18a-5p targets by TargetScan v7.2.

**TABLE S9** **|** Predicted miR-18a-3p targets by TargetScan v7.2.

**TABLE S10** **|** High-confidence miR-18a-5p targets in VEM resistance prioritized by TargetScore.

**TABLE S11** **|** High-confidence miR-18a-5p targets in CD8^+^ T cell tolerance prioritized by TargetScore.

**TABLE S12** **|** High-confidence miR-18a-3p targets in VEM resistance prioritized by TargetScore.

**TABLE S13** **|** High-confidence miR-18a-3p targets in CD8^+^ T cell tolerance prioritized by TargetScore.

**TABLE S14** **|** miR-18a-5p activity scores in targeted therapy patients (VEM resistance-associated genes).

**TABLE S15** **|** miR-18a-5p activity scores in immunotherapy patients (CD8^+^ T cell tolerance-associated genes).

**TABLE S16** **|** miR-18a-3p activity scores in targeted therapy patients (VEM resistance-associated genes).

**TABLE S17** **|** miR-18a-3p activity scores in immunotherapy patients (CD8^+^ T cell tolerance-associated genes).

**TABLE S18** **|** CIBERSORT ∆-immune cell proportions and ∆-miR-18a-5p scores in immunotherapy patients.

**TABLE S19** **|** CIBERSORT ∆-immune cell proportions and ∆-miR-18a-3p scores in immunotherapy patients.

**TABLE S20** **|** DEGs in A375-VR versus WT A375 cells.

**TABLE S21** **|** DEGs in A375-TR versus WT A375 cells.

**TABLE S22** **|** Clinical characteristics of 53 melanoma patients treated with targeted therapy or immunotherapy.

**TABLE S23** **|** miR-18a and hnRNP A1 expression levels across treatment modalities in melanoma patients.

**TABLE S24** **|** Primers used in the experiments.
